# Acetylation-dependent regulation of core spliceosome modulates hepatocellular carcinoma cassette exons and sensitivity to PARP inhibitors

**DOI:** 10.1038/s41467-024-49573-7

**Published:** 2024-06-18

**Authors:** Linmao Sun, Yufeng Liu, Xinyu Guo, Tianming Cui, Chenghui Wu, Jie Tao, Cheng Cheng, Qi Chu, Changyong Ji, Xianying Li, Hongrui Guo, Shuhang Liang, Huanran Zhou, Shuo Zhou, Kun Ma, Ning Zhang, Jiabei Wang, Yao Liu, Lianxin Liu

**Affiliations:** 1 Department of Hepatobiliary Surgery, Centre for Leading Medicine and Advanced Technologies of IHM, The First Affiliated Hospital of USTC, Division of Life Sciences and Medicine, University of Science and Technology of China, Hefei, 230001 Anhui China; 2 Anhui Provincial Key Laboratory of Hepatopancreatobiliary Surgery, Hefei, 230001 Anhui China; 3 Anhui Provincial Clinical Research Center for Hepatobiliary Diseases, Hefei, 230001 Anhui China; 4 Department of Gastrointestinal Surgery, The First Affiliated Hospital of USTC, Division of Life Sciences and Medicine, University of Science and Technology of China, Hefei, 230001 China; 5 Department of Endocrinology, The First Affiliated Hospital of USTC, Division of Life Sciences and Medicine, University of Science and Technology of China, Hefei, 230001 China

**Keywords:** Hepatocellular carcinoma, Acetylation, RNA splicing

## Abstract

Despite the importance of spliceosome core components in cellular processes, their roles in cancer development, including hepatocellular carcinoma (HCC), remain poorly understood. In this study, we uncover a critical role for SmD2, a core component of the spliceosome machinery, in modulating DNA damage in HCC through its impact on *BRCA1*/*FANC* cassette exons and expression. Our findings reveal that SmD2 depletion sensitizes HCC cells to PARP inhibitors, expanding the potential therapeutic targets. We also demonstrate that SmD2 acetylation by p300 leads to its degradation, while HDAC2-mediated deacetylation stabilizes SmD2. Importantly, we show that the combination of Romidepsin and Olaparib exhibits significant therapeutic potential in multiple HCC models, highlighting the promise of targeting SmD2 acetylation and HDAC2 inhibition alongside PARP inhibitors for HCC treatment.

## Abstract (web summary)

The relevance of spliceosome core components in cancer is less understood. Here the authors show SmD2, a core component of the spliceosome machinery, is under acetylation-dependent regulation, which could be targeted to enhance sensitivity to PARP inhibitors in hepatocellular carcinoma.

## Introduction

Hepatocellular carcinoma (HCC) is one of the leading causes of cancer death^1^. Nonetheless, there has been a lack of research into the potential roles of splicing processes in HCC. Dysregulation of splicing is a common trait in cancer^2^, which often shows considerable divergences in alternative splicing compared to normal tissues^3–5^. Gene mutations encoding core or regulatory spliceosomal components^6–8^, along with specific tumor-associated changes in RNA splicing factors^9^ have been associated with this phenomenon. However, advanced knowledge on how and to what extent spliceosomal machinery affects the development and progression of HCC remains insufficient.

The spliceosome is a complex composed of five small nuclear ribonucleoproteins (snRNPs) U1, U2, U4, U5 and U6, along with over 200 non-snRNP protein components that are responsible for almost all splicing reactions^10^. The maturation and nuclear importation of U snRNPs involves the assembly of Sm proteins SmB, D1, D2, D3, E, F and G onto the specific sequence (Sm site) of nuclear RNAs (snRNA)^11^. PRMT5-mediated methylation of SmB, D1 and D3 facilitates this process, which influences the levels of snRNPs and, consequently, alternative splicing decisions^12,13^. Currently, clinical trials investigating PRMT5 inhibitors for treatment of advanced MDS/AML and advanced solid tumors/hematological malignancies are in progress^14^. Although Sm proteins are essential in the splicing process, the mechanisms and functions of other components, such as SmD2, in tumors haven’t been thoroughly investigated.

PARP inhibitor (PARPi) was utilized for synthetic lethality strategy targeting DNA damage repair with the emergence of therapy for various cancers, especially *BRCA1/2*-deficient cancers^15^. However, single PARPi therapy in *BRCA*-WT tumors appears to be limited in effectiveness. Thus, many investigations have been carried out to examine the potential of PARPi in combination with other therapeutic approaches in *BRCA*-WT tumors^16,17^.

Lysine acetylation of non-histone proteins can compete with ubiquitination as well as enhance ubiquitylation, ultimately influencing protein stability^18,19^. Notably, TIP60-regulated acetylation of DNA (cytosine-5)-methyltransferase 1(DNMT1), mainly expressed in the nucleus, stimulates its ubiquitylation by E3 ubiquitin-protein ligase UHRF1, targeting DNMT1 for proteasomal degradation^20^. SmD2 is also a nuclear protein, but its regulatory mechanism, functional effects, and acetylation status remain unclear.

In this study, we investigate the acetylation status of SmD2 and its impact on DNA damage repair through regulating the splicing process in HCC. Our findings suggest SmD2 as a potential target for therapeutics in HCC. Additionally, combining an inhibitor of histone deacetylase (HDAC) with a PARP inhibitor serves as a therapeutic approach for HCC based on spliceosome and alternative mechanism perspectives.

## Results

### Identification of Smd2 as a potential biomarker for HCC diagnosis and prognosis

**Fig. 1:**
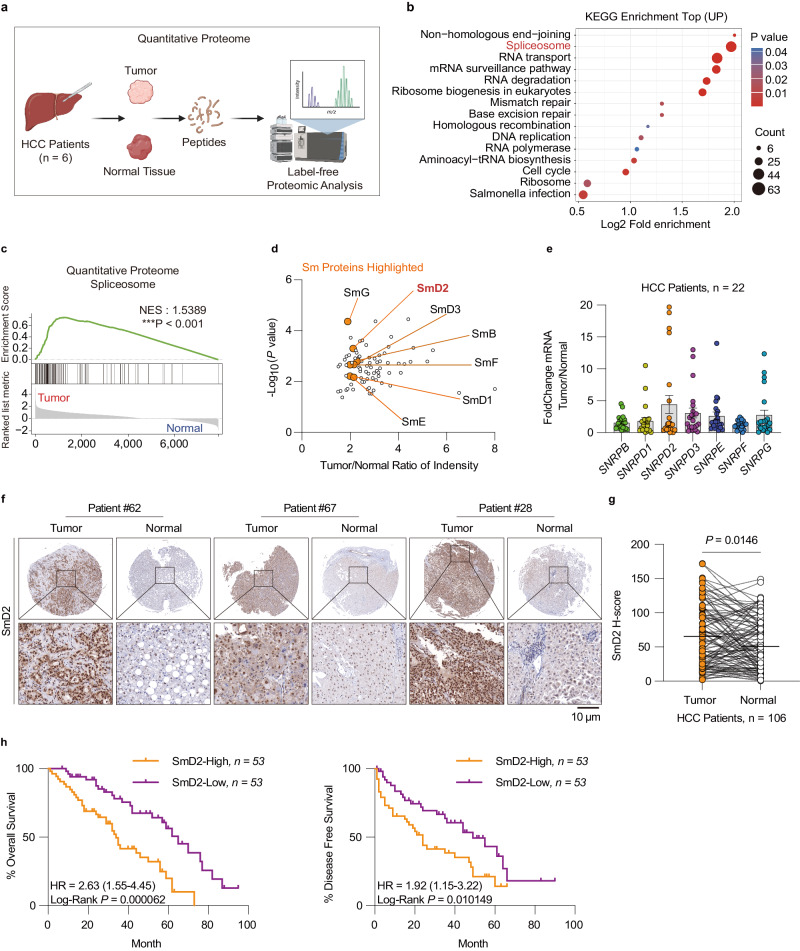
High expression of SmD2 is correlated with poor prognosis in HCC. **a** Schematic representation of the label-free quantitative proteomics analysis workflow performed on liver tumor and adjacent non-tumor tissue samples from six HCC patients. **b** the Kyoto Encyclopedia of Genes and Genomes (KEGG) pathway enrichment analysis of the proteomics revealed the Spliceosome pathway to be the rank 2 significantly upregulated pathway. two-sided hypergeometric test, with *P* values adjusted for multiple comparisons using the Benjamini–Hochberg False Discovery Rate (FDR) method. **c** Gene Set Enrichment Analysis (GSEA) was used to analyze the Spliceosome gene signatures in the proteomic data of tumor and normal tissues; the Normalized Enrichment Score (NES) was used to measure the degree of enrichment. one-sided permutation test. **d** Volcano plot highlighting the significant upregulation of Sm proteins in HCC tumors (orange dots) with respect to normal tissue, with SmD2 showing the most pronounced increase in tumor/normal ratio of intensity. **e** Analysis of mRNA expression differences of 7 Sm proteins in tumor and normal tissues from 22 HCC patients. mean values ± SEM. **f** Representative immunohistochemical staining for SmD2 in two tissue microarrays (TMAs) of 106 HCC patients, showcasing an increased SmD2 expression in tumor regions compared to normal tissue. **g** Quantitative analysis of SmD2 staining intensity in two TMAs from 106 HCC patients, two-sided t-test. **h** Kaplan–Meier survival analysis using the median expression level of SmD2 to stratify cases into high and low SmD2 expression groups. Overall and disease-free survival curves are generated for each group and compared using the log-rank test. Analysis of two TMAs encompassing 106 HCC patients reveals that high SmD2 expression is associated with a poorer prognosis. Figure 1a created with BioRender.com, released under a Creative Commons Attribution-NonCommercial-NoDerivs 4.0 International license. Source data are provided as a Source Data file.

An unbiased label-free quantitative proteome was employed in tumor tissues and corresponding normal liver tissues from six patients with HCC to identify vital players in the disease progression (Fig. 1a). Upon analysis of upregulated proteins in the tumor, the Kyoto Encyclopedia of Genes and Genomes (KEGG) pathway enrichment analysis revealed that the spliceosome pathway is rank 2 among the top enriched pathways (Fig. 1b). The finding was further confirmed by gene set enrichment analysis (GSEA), indicating the indispensability of splicing progression in HCC development (Fig. 1c). The Sm proteins, which are key components of the U snRNPs, immediately caught our attention among the upregulated spliceosome proteins (Fig. 1d, Supplementary Fig. 1a). This is due to the essential nature of the Sm-snRNA ring, comprising seven Sm proteins and U snRNA, in U snRNP maturation^21^.

Moreover, the TCGA-LIHC dataset also showed that seven Sm proteins were highly expressed (Supplementary Fig. 1b), and elevated expression of *SNRPB*, *D1*, *D2*, *F*, and *G* (which encode SmB, D1, D2, F, and G) correlated with poor prognosis (Supplementary Fig 1c–i). Subsequently, we expanded our samples to 22 human HCC tissues, and our qPCR analysis revealed that SmD2 was the most significantly upregulated protein among the seven Sm proteins (Fig. 1e). Immunohistochemistry analysis of two tissue microarrays (TMAs) consisting of 105 HCC samples indicated that SmD2 was upregulated in tumor tissues compared to normal tissues (Fig. 1f, g). Additionally, the prognostic analysis showed that elevated levels of SmD2 were associated with poor prognosis in individuals with HCC in the TMAs (Fig. 1h). These findings suggest that SmD2 may serve as a potential biomarker for HCC diagnosis and prognosis, and further exploration is warranted.

### SmD2 depletion impairs HCC tumorigenesis

**Fig. 2:**
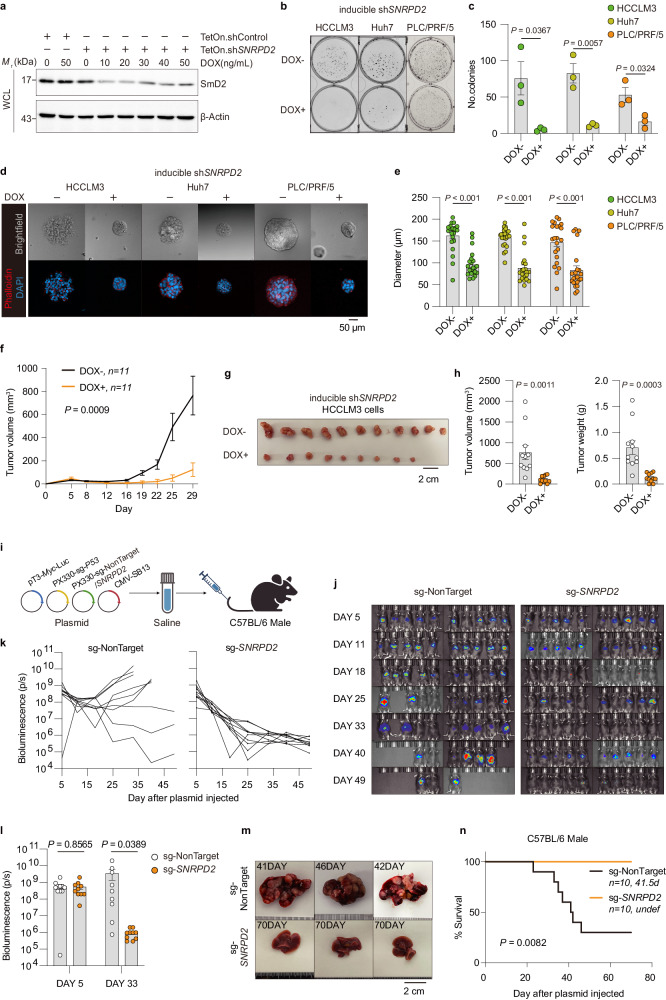
Impact of SmD2 deficiency on HCC progression. **a** Immunoblot demonstrating the effects of doxycycline (DOX) titration on EZ-Tet-pLKO-sh*SNRPD2* cells, with 72 hours of treatment followed by cell lysis. The experiment was repeated three times with similar results. WCL, Whole Cell Lysate. **b** Representative images of colony-formation assays of uninduced or DOX-induced sh*SNRPD2* expression across three HCC cell types. **c** Quantification of the colony formation assay (*n* = 3 biologically independent experiments) shown in **b** for three different cell lines. two-sided t-tests. **d** Representative confocal images of spheroid formation assays with DOX-induced *SNRPD2* knockdown in HCC cell lines, stained with Phalloidin (to stain the cytoskeleton) and DAPI (to stain nuclei). **e** Quantitative analysis of spheroid diameters (*n* = 22 biologically independent samples) from the assay in **d**, showing a significant decrease in spheroid size with *SNRPD2* knockdown across all three cell lines. two-sided t-tests. **f** Tumor growth of inducible sh*SNRPD2* HCCLM3 cells in male nude mice, with doxycycline (20 mg/kg) orally administered every other day to induce sh*SNRPD2* expression, beginning on day 7. Two-way ANOVA. **g** Representative images of tumor volumes from HCCLM3-harboring mice sacrificed on day 29. **h** Quantitative analysis of tumor (*n* = 11) volume and weight from (**g**). two-sided t-tests. **i** Schematic illustrating transposon- and CRISPR-Cas9-based hydrodynamic injection of vectors into mice via the tail vein to generate liver tumors resembling human HCC. The transposon-based vector overexpressing *MYC* can express luciferase. **j** Bioluminescence images of C57BL/6 mice (*n* = 10) at indicated time points after plasmid injection, indicating tumor burden over time. **k** Individual bioluminescence trajectories for each mouse over time, comparing non-targeting control to sg-*SNRPD2*. **l** Bioluminescence signal at indicated time points post plasmid injection in C57BL/6 mice (*n* = 10). two-sided t-tests. **m** Representative images of livers from **j**. **n** Kaplan–Meier survival curve of C57BL/6 mice treated as in **j**. log-rank test. Data are presented as mean values ± SEM. Figure 2i created with BioRender.com, released under a Creative Commons Attribution-NonCommercial-NoDerivs 4.0 International license. Source data are provided as a Source Data file.

To evaluate the role of SmD2 in HCC, we first conducted knockdown experiments in HCCLM3 cells. As a result, we observed a significant decrease in cell growth (Supplementary Fig. 2a–c). To further verify the result without the bias caused by the transfection process, we established a Tet-On system to induce the knockdown of *SNRPD2* (Fig. 2a). Remarkably, the induction of sh*SNRPD2* via Dox treatment led to reduced cell proliferation in HCC cells by colony-formation (Fig. 2b, c) and in a three-dimensional (3D) cell-culture system (Fig. 2d, e), which better mimics an in vivo setting.

Additionally, we utilized the inducible sh*SNRPD2* HCCLM3-harboring mice approach to assess the effects of SmD2 depletion on tumor development. Our results demonstrated that *SNRPD2* knockdown strongly reduced the tumor growth rate (Fig. 2f–h). However, it is essential to consider that subcutaneous xenograft models cannot reproduce the entire liver microenvironment accurately. Hence, we employed a previously described *p53* knockout and *MYC* overexpression system in hepatocytes to induce murine HCC and characterize the impact of SmD2 on HCC development^22^. Via hydrodynamic injection of plasmids into 5-week-old C57BL/6 male mice, we observed equivalent luciferase expression in the livers (Fig. 2i, Supplementary Fig. 2d), as measured by bioluminescence imaging on day 5 (Fig. 2j–l), indicating similar injection efficiency and expression levels in both groups. The experimental group injected with pX330-sg-*SNRPD2* of mice with *MYC; p53*^*-/-*^ HCC showed a dramatic decrease in luciferase expression at day 33 after plasmid injection compared to that in control mice (injected with pX330-sg-*NonTarget*) (Fig. 2l). Significantly, no tumors developed in any experimental mice within 70 days, while most control mice (7/10) developed tumor burden, leading to death with a median survival time of 41.5 days (Fig. 2m, n). Interestingly, when we knocked down *SNRPD2* in the normal hepatic stellate LX-2 cell line, we observed no impact on its cell viability (Supplementary Fig. 2e). This finding aligns with previous research conducted in normal lung fibroblasts^23^, indicating that while SmD2 is indispensable for tumor cells, targeting SmD2 seems to have little influence on the vitality of normal cells. Taken together, our data confirm that SmD2 depletion impairs HCC tumorigenesis both in vitro and in vivo, indicating its importance in the progression of HCC.

### SmD2 regulates DNA damage response

**Fig. 3:**
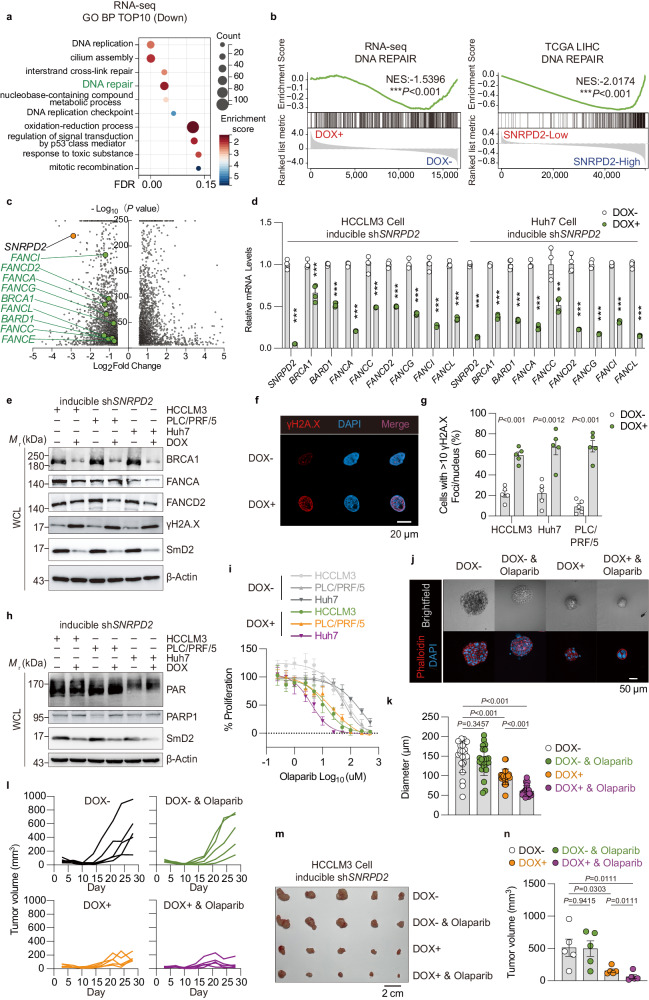
SmD2 depletion enhances the accumulation of DNA damage and sensitivity to PARP inhibitors in HCC. **a** Gene Ontology analysis of genes (*n* = 2636, *p* < 0.05) downregulated by inducible sh*SNRPD2* Knockdown in HCCLM3 cells, compared to control cells. **b** Gene Set Enrichment Analysis (GSEA) indicating that DNA repair pathways are significantly downregulated in HCC cells with *SNRPD2* knockdown (DOX+) and in HCC samples from The Cancer Genome Atlas (TCGA) with low expression of *SNRPD2* (coding SmD2). **c** Volcano plot of differentially expressed genes after DOX-induced *SNRPD2* knockdown, with |Log2 (fold change)| = 0.5 and –log10(P-value) = 6 as thresholds; genes with y > 250 are shown as y = 250. Genes involved in DNA repair pathways such as *FANCA, FANCD2, BRCA1* are notably downregulated. **d** Quantitative RT-PCR validation of RNA-seq data showing reduced mRNA levels of several DNA repair genes with *SNRPD2* knockdown. (*n* = 4 independent experiments). **e** Immunoblot showing BRCA1, FANCA, FANCD2 and γH2A.X expression in inducible sh*SNRPD2* cells. The experiment was repeated three times with similar results. **f** DNA damage accumulation with or without silencing of *SNRPD2* detected using γH2A.X immunostaining. **g** Percentage of cells with ≥10 γH2A.X foci/nucleus, analyzed in random cells (*n* = 5 independent experiments). **h** Levels of PARylation and PARP1 expression measured by immunoblot in inducible sh*SNRPD2* cells. The experiment was repeated three times with similar results. **i** Olaparib dose-response curves on inducible sh*SNRPD2* HCC cell lines over 72 hours (*n* = 4 biologically independent samples). **j** Representative 3D culture plot of transfected inducible sh*SNRPD2* HCCLM3 cells after DOX and Olaparib (IC20) treatment. **k** Diameter of the cell spheres (*n* = 22 biologically independent samples) counted from **j**. **l** Inducible sh*SNRPD2* HCCLM3 cells were seeded into male nude mice. Doxycycline (20 mg/kg, orally, daily) and Olaparib (50 mg/kg, intraperitoneally, daily) were administered from day 10. Each line represents an individual tumor. **m** Tumor volume reduction in HCCLM3 xenografts after inducible *SNRPD2* knockdown and further inhibition by Olaparib treatment, with representative images. **n** Statistical analysis of tumor volume data from (**m**) (*n* = 5). Mean values ± SEM. Two-sided t-tests were used to determine statistical significance, **P = 0.0022, and ***P < 0.001. Source data are provided as a Source Data file.

To further elucidate the physiological mechanism of SmD2 on HCC, we performed RNA-sequencing (RNA-seq) analysis to compare gene expression profiles between inducible *SNRPD2* knockdown and non-induction (as control) HCCLM3 cells. Results showed that SmD2 deficiency in HCC cells downregulated genes associated with DNA repair mechanisms, such as homologous recombination repair and interstrand cross-link repair (Fig. 3a–c, Supplementary Fig. 3a). In agreement with our RNA-seq findings, the GSEA of TCGA-LIHC database showed that DNA repair genes were enriched in human *SNRPD2*-High HCC samples compared to *SNRPD2*-Low samples (Fig. 3b). Subsequently, we verified the decreased expression of DNA repair genes in multiple HCC cell lines and xenografts (Fig. 3d, e, Supplementary Fig. 3b). Analysis of a TCGA-LIHC dataset also suggested that *BRCA1*, *FANCA*, *FANCD2*, *FANCI*, and *FANCG* mRNA levels were positively correlated with *SNRPD2* mRNA levels in human HCC samples (Supplementary Data Fig. 3c). These data indicate that SmD2 knockdown may trigger a DNA damage response (DDR) through the downregulation of DNA repair genes in HCC. To assess DDR induction, we analyzed the formation of phosphorylated H2A.X (γH2A.X) foci as a marker for double-stranded DNA breaks (DSBs) and identified a significant increase in γH2A.X-positive cells after *SNRPD2* knockdown in HCC cells (Fig. 3e–g, Supplementary Fig. 3b), which further supports our conclusion that SmD2 loss induces DDR.

### Synergistic effects of SmD2 knockdown and pharmacological inhibition of PARP1

The synthetic-lethal relationship between BRCA1/2 and PARP1 has been extensively researched, with PARPi swiftly gaining clinical application^24^. PARPi impedes tumorigenesis by blocking PARP1 at the site of DNA lesions, inducing DNA-protein crosslinks that trigger replication fork collapse, and subsequently accumulate DSBs during S-phase in the cell cycle^15,25–27^. Via GSEA of RNA-seq, we found that *SNRPD2* knockdown did not affect the base excision repair pathway and single-strand break repair pathway mediated by PARP1 (Supplementary Fig. 3a). Besides, knocking down *SNRPD2* did not affect the expression or PARylation of PARP1 in HCCLM3 and Huh7 cells, yet it resulted in a slight increase in both the expression and PARylation of PARP1 in PLC/PRF/5 cells (Fig. 3h). Given that PARPi (particularly the FDA-approved Olaparib, Rucaparib, Niraparib and Talazoparib) limit auto PARylation and PARP1 release from the site of damage, hindering with the catalytic cycle of PARP1^28–30^, we hypothesized that PARPi might synergize with SmD2 depletion to restrict HCC growth. To verify this hypothesis, we treated inducible sh*SNRPD2* HCC cells with Olaparib, Rucaparib, Niraparib and Talazoparib. We found that *SNRPD2* knockdown substantially increased the cytotoxicity of PARPi (Fig. 3i, Supplementary Fig. 3d). Additionally, we confirmed this observation regarding Olaparib using 3D culture assays and HCCLM3 xenografts (Fig. 3j–n). However, our results suggest that PARPi alone might be insufficient for treating HCC, consistent with previous studies^31,32^. Our findings indicate that pharmacological inhibition of PARP1 impedes tumorigenic growth in HCC cell lines exhibiting SmD2-deficiency.

### Smd2 depletion promotes mis-splicing and degradation of DNA repair genes

**Fig. 4:**
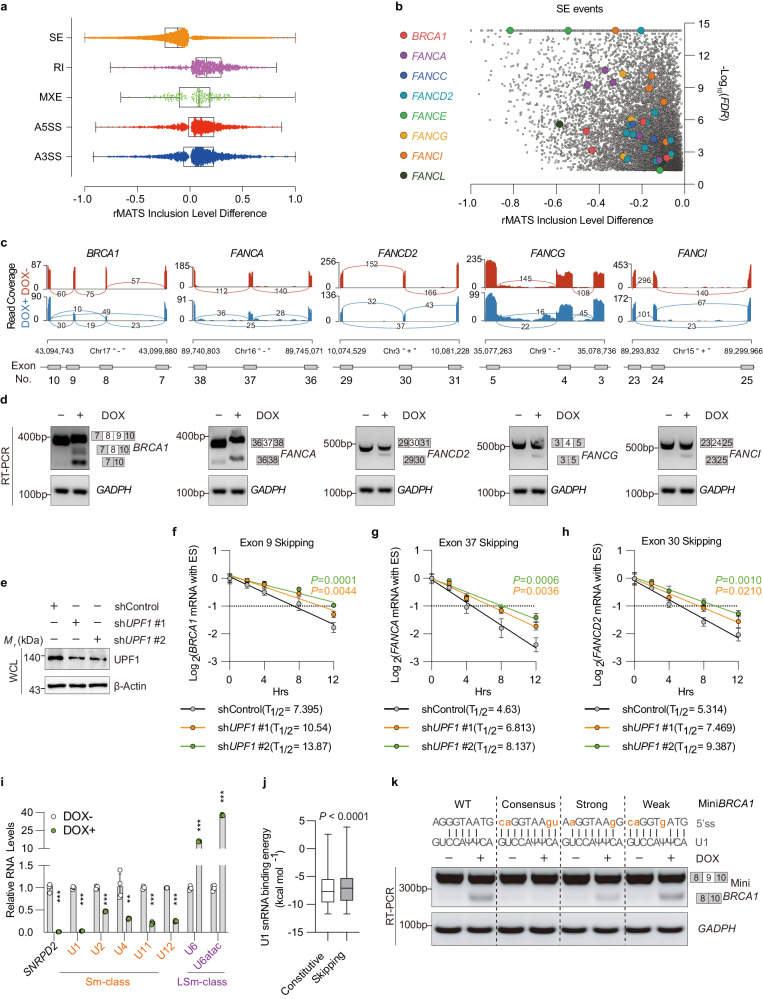
Analysis of SmD2-mediated alternative splicing in DNA repair genes. **a** Differences in rMATS inclusion values in inducible sh*SNRPD2* HCCLM3 cells (*n* = 3/group biologically independent samples) after doxycycline (DOX) treatment are presented as median values, where the center line in each box represents the median, the edges of the boxes represent the first (25th percentile) and third (75th percentile) quartiles, and the whiskers extend to the minimum and maximum values. A5SS, alternative 5’ splicing site; A3SS, alternative 3’ splicing site; RI, retained introns; SE, skipped exons; MXE, mutually exclusive exons.” **b** A half-volcano plot showing SE events in DNA repair genes is highlighted, where an inclusion value difference < −0.01 and –log10(*P*-value) = 2 were used as thresholds, and events with y > 16 are shown as y = 16. **c**, Visualizations of RNA-seq profiles (sashimi plots) of DNA repair genes. **d** RT-PCR validation of alternative splicing events in *BRCA1*, *FANCA*, *FANCD2*, *FANCG*, and *FANCI* genes, with *GADPH* as a loading control. Numbers in white boxs indicate exon skipping. The experiment was repeated three times with similar results. **e** Immunoblot analysis of SmD2 depletion HCCLM3 cells transduced with sh*UPF1*. The experiment was repeated three times with similar results. Half-life of *BRCA1* transcripts with exon 9 skipping (**f**), *FANCA* transcripts with exon 37 skipping (**g**), and *FANCD2* transcripts with exon 30 skipping (**h**) were measured by qPCR (*n* = 4 biologically independent samples). **i** qPCR examining the changes of spliceosomal snRNAs in HCCLM3 cells after DOX-induced SmD2 knockdown (*n* = 4 biologically independent samples). **j** U1 snRNA binding energy for 5’ splicing sites (5’ss) in skipped exons (*n* = 9580) influenced by *SNRPD2* knockdown compared to constitutive exons (*n* = 200,234). The central line in each box indicates the median of the dataset, with the box edges representing the 25th and 75th percentiles. The whiskers span from the minimum to the maximum data points, without any points classified as outliers. **k** Inducible sh*SNRPD2* HCCLM3 cells were transfected with wild-type (WT) mini*BRCA1* or with minigenes harboring mutations in the 5′ss, which were analyzed with RT–PCR assays to quantify the percent exon inclusion. Mutations that strengthen the 5′ss reduce the effects of *SNRPD2* knockdown on mini*BRCA1* SE. (ψ) Pseudouridine. The experiment was repeated three times with similar results. Mean values ± SD. Two-sided t-tests, **P = 0.002288, and ***P < 0.001. Source data are provided as a Source Data file.

As SmD2 is a core component of the splicing complex, we employed transcriptome data to study splicing changes induced by this protein. We used replicates of multivariate analysis of transcript splicing (rMATS) and found that *SNRPD2* knockdown cells showed a heightened number of alternative splicing events compared to non-induced cells (Fig. 4a). Notably, transcripts containing skipped exons (SEs) exhibited a significant and remarkable upregulation among various forms of splicing affected by sh*SNRPD2* knockdown (Fig. 4a). These SE events contained several DNA repair genes regulated by SmD2, such as *BRCA1* and Fanconi anemia subtype (*FANC*) genes, which we validated through RT-PCR (Fig. 4b–d).

Pre-mRNA alternative splicing can regulate gene expression levels by coupling with nonsense-mediated mRNA decay (NMD)^33^. To determine whether *SNRPD2* knockdown triggered degradation of these cassette exons by NMD, we measured the half-life of the exclusion isoform of *BRCA1* and *FANC* genes in SmD2-deficient HCCLM3 cells transfected with either a control or anti-*UPF1* shRNA – UPF1 is required for NMD following transcriptional shut-off induced by actinomycin D^34^. Our results indicated a significant increase in the mRNA half-life of the exclusion isoform of DNA repair genes, namely *BRCA1* and *FANC* genes, consequent to *UPF1* knockdown, providing evidence of NMD involvement (Fig. 4e, h). Thus, our data indicate that the specific isoform of *BRCA1* and *FANC* genes promoted by *SNRPD2* knockdown undergoes NMD.

### Characteristics of SmD2-regulated exons

In investigating the broad impact of SmD2 on exon skipping, we meticulously evaluated the traits of exons regulated by SmD2. Our analysis began with reassessing MaxEntScan scores for the 3’ acceptor splicing sites (3’ SSs) and donor splicing sites (5’ SSs) of impacted exons, alongside scores for unaffected alternative (reference) exons and constitutive exons (Supplementary Fig. 4a, b). We discovered that exons skipped due to sh*SNRPD2* knockdown presented significantly weaker MaxEnt scores at both splice sites compared to their constitutive and reference counterparts. Our study further delved into comparing exon length and GC content among affected, constitutive, and reference alternative exons. Affected exons were found to be shorter and exhibited a slight reduction in GC content frequency, indicating potential factors influencing exon skipping (Supplementary Fig. 4c–f). However, analysis of the polypyrimidine tract (PPT) showed no notable differences in score, length, or GC content across groups (Supplementary Fig. 4g–k), though an increased distance from the PPT to the 3’ splice site was observed in skipped exons (Supplementary Fig. 4l). Additionally, we analyzed CG content at specific positions within the exon regions near splice sites (Supplementary Fig. 4m), noting an increase in CG content at the −1 position but a decrease at the −2 position of 5’ splice sites in affected exons (Supplementary Fig. 4n–p). No significant correlation was found in CG content at various positions of 3’ splice sites (Supplementary Fig. 4q–s).

This concise examination underscores the influence of SmD2 on exon characteristics, such as splice site strength, exon length, and nucleotide composition, which collectively contribute to the regulation of exon skipping.

### SmD2 depletion affects exon skipping via the 5’ splice site-U1 snRNP interaction

Previous studies indicate concordance between spliceosomal snRNA levels measured in total cellular RNA and those in snRNPs isolated by anti-Sm antibodies^35^. The availability of a large pool of stable Sm proteins enables the rapid assembly with less abundant snRNAs in the cytoplasm^36^. As snRNA binding with the Sm site is essential for maintaining snRNP stability^37^, we hypothesized that the reduced levels of SmD2 could potentially affect the snRNA levels. Our investigation, consistent with prior research on SmB and SmD1^38^, disclosed that SmD2 silencing decreased Sm-class snRNAs rather than LSm-class snRNAs in various HCC cell lines (Fig. 4i, Supplementary Fig. 4t, u). Notably, our study exposed a marked reduction in U1 snRNA expression across all three HCC cell lines, potentially owing to interactions between the last two nucleotides of the U1 Sm site with both SmD2 and SmF^39^.

The initiation of spliceosome assembly involves recognition and definition of the 5’SSs by the U1 snRNP, with competition between 5’SSs and 3’SSs during splicing complex assembly leading to alternative exon skipping in final mRNA products^40^. Our data identify a robust functional link between SmD2 and U1 snRNA, where 5’SSs of skipped exons that responded to SmD2 loss exhibited weaker strength (Supplementary Fig. 4a) and lower match to the U1 snRNA consensus sequence compared to constitutive exons (Fig. 4j). Furthermore, our introduction of 5′SS mutations into *miniBRCA1* revealed that increasing the strength of the 5’SS substantially reduced the impact of SmD2 depletion in regulating exon skipping (Fig. 4k).

Altogether, these findings suggest the critical contribution of the 5’SS sequence in linking SmD2 with exon skipping regulation, with reduced interaction rates between 5’SS and U1 snRNP being a probable underlying cause for the observed skipped exon events in response to SmD2 depletion.

### SmD2 is acetylated at Lys 8 by p300

**Fig. 5:**
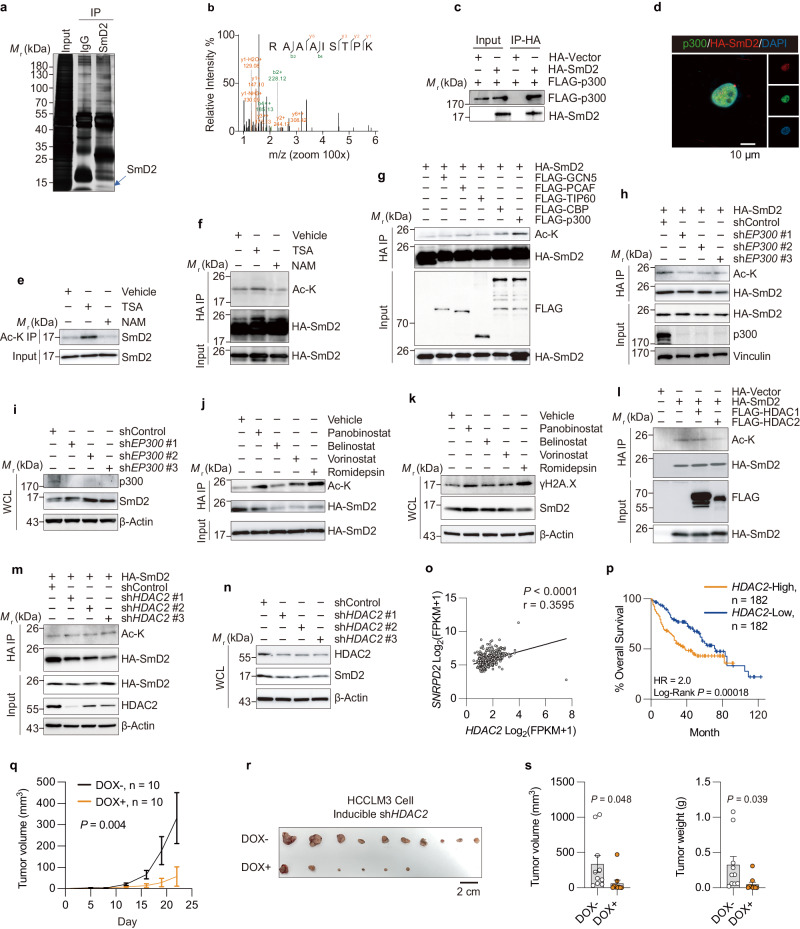
Mechanism of p300/HDAC2-mediated regulation of SmD2. **a** Immunoprecipitation coupled with mass spectrometry analysis was used to identify SmD2-interacting proteins in HCCLM3 cells (blue arrow indicates SmD2 signal). **b** Mass spectrometry spectrum showing the peptide of p300. **c** Co-immunoprecipitation of exogenous SmD2 and p300 in HEK293T cells after transfection with the indicated plasmids. **d** Immunofluorescence analysis to detect colocalization of SmD2 and p300 in HCCLM3 cells. The experiment was repeated three times with similar results. **e** Immunoblot to detect the acetylation of endogenous SmD2 in HCCLM3 cells treated with the SIRT inhibitor (5 mM NAM) or the HDAC inhibitor (1 μM TSA) overnight. Ac-K, acetylated lysine. **f** Immunoblot to detect the acetylation of exogenous HA-SmD2 in HEK293T cells treated with TSA or NAM. **g** Immunoblot of input and anti-HA immunoprecipitates (IPs) derived from HEK293T cells transfected with HA-SmD2 and Flag-tagged GCN5, PCAF, TIP60, CBP, or p300. **h** Immunoblot of input and anti-HA IPs derived from HEK293T cells transduced with HA-SmD2 and shp300. **i** Immunoblot of SmD2 changes from HCCLM3 cells after *EP300* depletion. **j** Immunoblot to detect the acetylation of exogenous SmD2 from HCCLM3 cells treated with FDA-approved HDAC inhibitors overnight. **k** Expression levels of SmD2 and γH2A.X from HCCLM3 treated with the HDAC inhibitors. WCL, whole cell lysate. **l** Immunoblot of input and anti-HA IPs transfected with HA-SmD2 and Flag-tagged HDAC1 or HDAC2. **m** Immunoblot of input and anti-HA IPs transduced with HA-SmD2 and sh*HDAC2*. **n** Immunoblot of SmD2 changes from HCCLM3 cells after *HDAC2* depletion. **o** Positive correlations of *SNRPD2* (encoding SmD2) mRNA levels with *HDAC2* mRNA levels in TCGA-LIHC dataset (*n* = 424, 374 of which were tumor samples and 50 adjacent normal tissues. r Pearson’s correlation coefficient, two-tailed t-distribution test). **p** Kaplan–Meier analysis of HCC patients from TCGA, stratified by *HDAC2* mRNA expression levels. Patients are categorized into two cohorts: those with *HDAC2* expression above the median and those with expression below the median. **q** Tumor growth of inducible sh*HDAC2* HCCLM3 cells in nude mice, with doxycycline orally administered, beginning on day 5. 2way ANOVA. **r** Representative images of tumor from HCCLM3-harboring mice. **s** Quantitative analysis of tumor (*n* = 10) volume and weight from (**r**). Mean values ± SEM. two-sided t-tests. Each Western blot was independently repeated three times. Source data are provided as a Source Data file.

Investigative efforts were undertaken to gain further insight into the molecular mechanisms governing SmD2 in HCC. Utilizing immunoprecipitation (IP) and liquid chromatography-tandem mass spectrometry (LC-MS/MS), a plethora of proteins were identified as putative interactors with SmD2 (Supplementary Data 2), including acetyltransferase p300 (Fig. 5a, b). The interaction between SmD2 and p300 was further confirmed via co-IP and immunofluorescence assays, highlighting SmD2’s potential for acetylation (Fig. 5c, d). This finding warrants further investigation to determine the broader implications of acetylation on SmD2 function in HCC.

To ascertain whether SmD2 is an acetylated protein, IP experiments were conducted to validate the acetylation status of both exogenously expressed and endogenous SmD2 (Fig. 5e, f). Ectopic expression of p300 significantly enhanced SmD2 acetylation, while CBP slightly increased it, unlike other acetyltransferases such as GCN5, PCAF, and TIP60 (Fig. 5g). Given the homology between CBP and p300, further knockdown of *EP300* (which encodes p300) inhibited this acetylation process (Fig. 5h), whereas *CREBBP* (which encodes CBP) had no effect (Supplementary Fig. 5a). These findings suggest that p300 could be the physiological acetyltransferase responsible for SmD2 acetylation. Subsequently, to identify the acetylation site on SmD2, we introduced substitution mutations of lysine (K) residues for arginine (R), based on the detection of 8 candidate sites highlighted by the IP-MS analysis. Our findings demonstrated that the K6R and K8R mutant lacked the ability to undergo acetylation mediated by p300 (Supplementary Fig. 5b–e).

As lysine acetylation can regulate non-histone protein stability^41^, we found *EP300* knockdown and treatment with c646, an inhibitor of p300, preventing degradation of SmD2 (Fig. 5i, Supplementary Fig. 5f). To delve deeper into which specific site might influence SmD2’s stability, we employed cycloheximide (CHX) chase analysis. This revealed a significant increase in the stability of SmD2 in K8R and double KR mutants compared to the wild-type and K6R following CHX treatment, highlighting the critical role of K8 deacetylation in safeguarding SmD2 from degradation (Supplementary Fig. 5g). Furthermore, our ubiquitination assays demonstrated a reduction in ubiquitination levels of K8R and double KR mutants relative to wild-type and K6R SmD2 (Supplementary Fig. 5h), supporting the notion that deacetylation at K8 may inhibit ubiquitin-mediated degradation, thereby augmenting SmD2’s stability. Notably, analyzing the TCGA-LIHC dataset suggested that levels of *EP300* were inversely correlated with those of *SNRPD2* in human HCC samples, and high *EP300* levels did not correlate with poor survival in HCC patients (Supplementary Fig. 5i, j). These data indicate that p300 is crucial in regulating SmD2 degradation through acetylation in HCC.

### HDAC2 specifically deacetylates SmD2

Given the link between SmD2 stability and acetylation, we hypothesized that targeting the deacetylase of SmD2 could be a viable strategy to halt HCC progression. The stimulation of SmD2 acetylation by trichostatin A (TSA) rather than nicotinamide (NAM) suggests the involvement of HDAC family deacetylases, not sirtuin deacetylase (SIRT) (Fig. 5e, f). We then treated HCC cells with four FDA-approved HDAC inhibitors: Panobinostat, Belinostat, Vorinostat, and Romidepsin. While each inhibitor increased SmD2 acetylation, Romidepsin—the selective HDAC1/2 inhibitor—was by far the most effective at facilitating both SmD2 acetylation and protein degradation compared to the other three pan-HDAC inhibitors (Fig. 5j, k). Importantly, Romidepsin treatment exhibited the highest cytotoxicity against HCC cells and significantly caused DNA damage accumulation. (Fig. 5k, Supplementary Fig. 6a). Our subsequent experiments confirmed that ectopic expression of HDAC2—not HDAC1—decreases SmD2 acetylation levels (Fig. 5l), while endogenous *HDAC2* depletion using shRNA increases SmD2 acetylation (Fig. 5m). Notably, *HDAC2* knockdown led to downregulation of SmD2 expression (Fig. 5n), and the TCGA dataset also revealed a positive clinical relevance between *SNRPD2* and *HDAC2* in human HCC (Fig. 5o, p). These data support a role for HDAC2 in the regulation of SmD2 and in HCC tumorigenesis.

Based on these findings, we propose that HDAC2 regulates HCC growth by modulating SmD2. To support this hypothesis, colony-formation assays utilizing three sh*HDAC2* sequences or Tet-ON system in HCCLM3 cells demonstrated a significant decrease in cell growth (Supplementary Fig. 6b–d). Moreover, inducible sh*HDAC2* HCCLM3 xenografts showed a considerably reduced tumor growth rate following *HDAC2* knockdown (Fig. 5q–s). In addition, *HDAC2* knockdown resulted in the downregulation of *BRCA1* and *FANC-type* genes causing DNA damage accumulation (Supplementary Fig. 6e–g). Replenishing SmD2 levels rescued not only the downregulated DNA repair changes but also restored cell growth in HCC cells (Supplementary Fig. 6f–h). Notably, *HDAC2* showed a strong positive correlation with these DNA repair genes in the TCGA-LIHC dataset (Supplementary Fig. 6i), which is consistent with the relationship observed with SmD2’s role in DNA repair pathways.

In summary, targeting HDAC2 and its modulation of SmD2 acetylation and expression represents a promising strategy for inhibiting HCC progression, with Romidepsin emerging as an effective therapeutic option.

### Romidepsin and PARPi combination therapy can further curb the multi-types of the HCC model

**Fig. 6:**
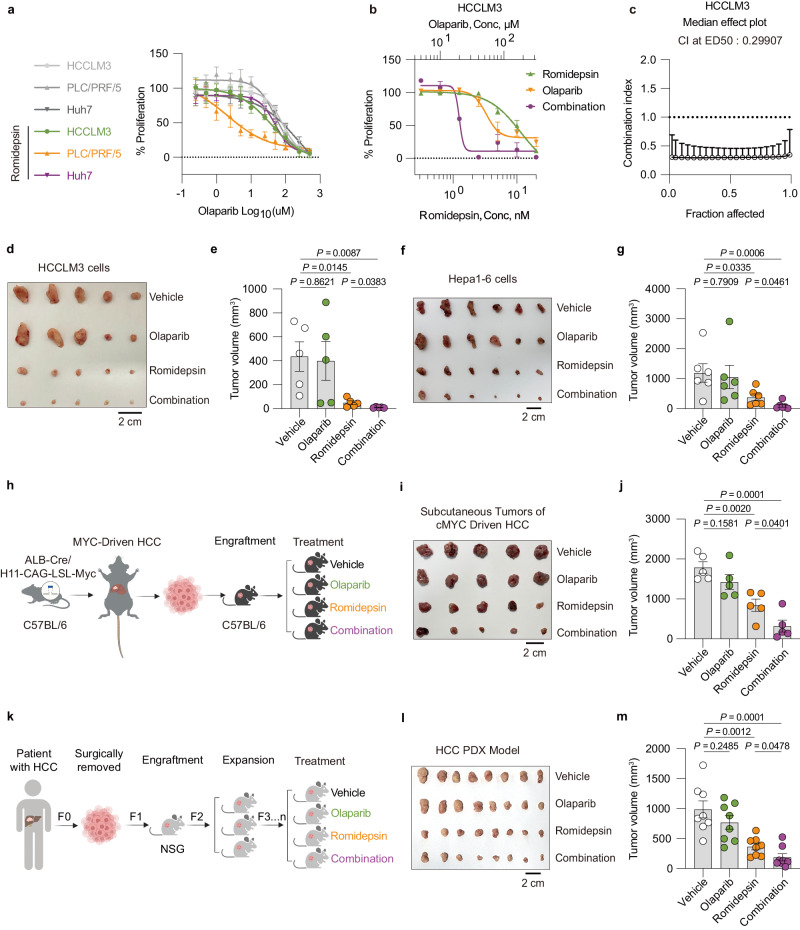
Romidepsin enhances the sensitivity of HCC to PARP inhibitors. **a** Dose-response curves for Olaparib in HCC cell lines following treatment with IC20 of Romidepsin (*n* = 4 biologically independent samples). **b** Synergistic dose-response curves for HCCLM3 cells treated with varying concentrations of Olaparib and Romidepsin (*n* = 4 biologically independent samples). **c** Combination index (CI) versus fraction affected for Olaparib and Romidepsin in HCCLM3 cells, derived from data in **b** (*n* = 4 biologically independent samples). **d** HCCLM3 tumors were dissected from the nude mice treated with vehicle, Olaparib, Romidepsin, or both (*n* = 5/group). **e** Quantitative analysis of tumor (*n* = 5) volume from **d**. **f** As **d**, but for Hepa1-6 tumor-bearing C57/BL6 mice (*n* = 6/group). **g** Quantitative analysis of tumor (*n* = 6) volume from **f**. **h** Schematic diagram of murine MYC-driven HCCs. After mating of H11^*LSL-Myc*^ mice harboring a CAG promoter-*loxp-STOP-loxp-Myc*-polyA conditional overexpression structure at the H11 locus with *Alb*-cre mice for two months, the spontaneous development of HCC was observed. Subsequently, these murine HCCs were transplanted into the subcutaneous tissues for treatment. **i** As **d**, but for MYC-driven HCC tumor-bearing C57/BL6 mice (*n* = 5/group). **j** Quantitative analysis of tumor (*n* = 5) volume from **i**. **k** Schematic diagram of human HCC PDX. **l** As **d**, but for HCC PDX tumor-bearing NSG mice (*n* = 8/group). **m** Quantitative analysis of tumor (*n* = 8) volume from **l**. Mean values ± SEM. Two-sided t-tests. Figures **h** and **k** created with BioRender.com, released under a Creative Commons Attribution-NonCommercial-NoDerivs 4.0 International license. Source data are provided as a Source Data file.

Since depletion of SmD2 has been shown to promote HCC sensitivity to PARPi, it was hypothesized that there may be synthetic lethality between HDAC2 and PARP inhibition. HCC cell lines were treated with Olaparib, Rucaparib, Niraparib, and Talazoparib under the pressure of Romidepsin and it was found that Romidepsin increased the cytotoxicity of PARPi (Fig. 6a, Supplementary Fig. 7a). We also tested for Olaparib synergy with Romidepsin in vitro, using the Chou-Talalay method^42^, and observed a Combination Index of <1, suggestive of a synergistic interaction between Olaparib synergy with Romidepsin (Fig. 6b, c, Supplementary Fig. 7b–e). Additionally, 3D-culture assays showed that Romidepsin decreased the proliferation of HCC cells under Olaparib treatment (Supplementary Fig. 7f, g).

To determine the effectiveness of combining Romidepsin and PARPi in vivo, we seeded HCCLM3 and Hepa1-6 tumors in nude mice and C57BL/6 mice, respectively. We began administering combination therapy with Romidepsin and Olaparib when tumor size stabilized across groups, approximately one week after the tumor became measurable (Supplementary Fig. 8a, c). As expected, Romidepsin monotherapy exhibited strong effects, whereas Olaparib monotherapy was not (Fig. 6d–g, Supplementary Fig. 8a–d). Simultaneous Romidepsin and Olaparib therapy led to remarkably decreased growth of both HCCLM3 and Hepa1-6 tumors, surpassing the effects of either drug alone, indicating significant potential for this combined therapy.

To better reflect the effect of the combination of Romidepsin and Olaparib on HCC, we used an H11^*LNL-Myc*^ mouse model that included a CAG promoter-*loxp*-*STOP*-*loxp-Myc*-polyA conditional overexpression structure in the H11 locus. This was developed by crossing H11^*LNL-Myc*^ heterozygous mice with *Alb*-cre transgenic mice to generate *Myc*/*Alb*-cre double-positive mice that spontaneously develop HCC after 2 months due to abnormal and activated Myc^43^, which is frequently altered in HCC patients^44^. To quantify the effectiveness of the combined use of Romidepsin and Olaparib, we subcutaneously transplanted Myc-driven HCC tumors in mice and administered the drugs accordingly (Fig. 6h). In the subcutaneous Myc-driven HCCs, we also observed an accordant result as HCCLM3 and Hepa1-6 tumors (Fig. 6i, j, Supplementary Fig. 8e, f). We further modeled the clinical efficacy of this combination therapy by generating patient-derived xenograft (PDX) models (Fig. 6k–m, Supplementary Fig. 8g, h). Furthermore, our data show that the combined administration of Olaparib and Romidepsin, within the tested doses and duration, does not adversely affect the weight or liver and kidney functions of the mice (Supplementary Fig. 8i–k). These findings support the potential clinical application of this combined therapy for the treatment of HCC.

## Discussion

Splicing factors have been the focus of most studies on splicing in HCC^45^, with limited attention given to the core spliceosomal machinery. In our study, we identify a role of the core component in regulating DNA damage in HCC through molecular links between SmD2 and abnormal *BRCA1/FANC* splicing and expression. Our findings explain why SmD2-regulated exon skipping influences DNA damage, as observed in embryogenesis^46^. Specifically, the interaction between U1 snRNP and 5’ splice sites determines the splicing process outcome regulated by SmD2.

Further exploration in our research demonstrates that depleting SmD2 significantly suppressed HCC tumorigenesis, and this effect was further enhanced by combining it with a PARP inhibitor. While HCC is not commonly associated with HR deficiency or mutations in *BRCA1/2*, our study reveals that knocking down *SNRPD2* can decrease BRCA1 expression, thereby strengthening this connection. Interestingly, the reduction of SmD2 does not affect PARP1 expression and activity in HCCLM3 and Huh7 cell lines. However, in PLC/PRF/5 cells, there is a moderate increase in PARylation and PARP1 expression, likely reflecting a cell line-specific compensatory response within the DNA damage pathways^47,48^. This suggests that targeting both spliceosomal components and DNA repair mechanisms can synergistically reduce tumor growth. Such a strategy not only addresses multiple aspects of tumor biology but also potentially circumvents the issue of drug resistance. Our results indicate that while SmD2 depletion alone offers considerable anti-tumor benefits, its combination with PARP inhibition provides a more comprehensive approach to cancer treatment. This underscores the importance of multi-targeted therapies in cancer, advocating for further research into combining splicing factor inhibitors with other agents to improve cancer treatment outcomes.

Additionally, we discovered that p300-mediated acetylation of lysine residue 8 on SmD2 leads to its degradation, while HDAC2 deacetylates SmD2, promoting its stability. Acetylation-dependent protein stability often inhibits proteasome-mediated degradation by preventing protein ubiquitylation, possibly due to acetylation and ubiquitylation competing for the same lysine residues^41^. Like other post-translational modifications, acetylation alters protein properties by transferring an acetyl group to lysine residues, potentially promoting degradation by attracting various protein complexes. For instance, acetylation at K420 and K435 enhances p62’s affinity for ubiquitin by disrupting UBA dimerization^49^. Our studies show that SmD2 acetylation at K8 increases its ubiquitination. These findings imply that targeting Sm proteins could go beyond the current use of PRMT5 inhibitors^2^.

Importantly, Our research validates the therapeutic potential of FDA-approved Romidepsin when combined with Olaparib against multiple HCC models. There have been reports on the combined use of HDAC inhibitors and PARP inhibitors in tumors, with the intrinsic mechanisms involved being multifaceted. For instance, HDAC inhibitors can also facilitate DNA damage through the production of Reactive Oxygen Species (ROS)^50^, effectively bridging the connection to the DNA damage targeted by PARP inhibitors^51^. HDAC inhibitors and PARP inhibitors can also downregulate transcription factors to reduce the tumor’s growth. For example, the HDAC inhibitor suberohydroxamic acid (SBHA) and the PARP inhibitor AG-014699 have been shown to inhibit the function of CHD4 in EpCAM^+^ liver cancer stem cells^52^, thereby suppressing tumor growth. Similarly, research in cutaneous T-cell lymphoma has highlighted how the combination of Talazoparib and Romidepsin can induce Blimp–1–mediated p53-dependent apoptosis^53^. Building on these insights, Louise Ramos et al. developed the dual-target inhibitor KT-3283, showcasing superior efficacy over the combination of Olaparib and Vorinostat in Ewing sarcoma^54^. Diverging from these approaches, our research introduces a perspective by examining this therapeutic strategy through the lens of the spliceosome.

Romidepsin and Olaparib, being FDA-approved drugs, offer a dependable safety profile. In clinical trials, increases in serum enzymes with Romidepsin alone were usually temporary and minor, with rare need for dose adjustments^55^. Likewise, for Olaparib, only 4% of patients experienced elevated serum transaminases, and a mere 1% or less saw levels exceeding five times the upper limit of normal^56^. Our study also demonstrated through toxicity experiments in various animal models of HCC that the combined use of Olaparib and Romidepsin at preclinical therapeutic concentrations is non-toxic. Our results support that such combination therapies could provide safe and effective treatment options by targeting the RNA splicing process.

In conclusion, our study highlights the critical regulatory role of SmD2 in alternative splicing events and their impact on DNA damage pathways, underscoring their potential as a promising therapeutic avenue for HCC treatment. Moreover, our results suggest that combining HDAC2 inhibition with PARPi could offer effective strategies against HCC.

## Methods

The research methodologies adhered to the principles outlined in the World Medical Association’s Declaration of Helsinki and its subsequent amendments. Furthermore, the Ethics Committee of The First Affiliated Hospital of the University of Science and Technology of China conducted a review and granted approval for this research.

### Cell culture, transfection, and virus infection

HCCLM3, Huh7, PLC/PRF/5, Hepa1-6, LX-2 and HEK293T cells were cultured in the DMEM (Sigma), which contained 10% FBS (Sigma), 100 U penicillin (Gibco) and 100 ug ml^−1^ streptomycin (Gibco). PLC/PRF/5 and Huh7 were purchased from Accegen. Hepa1-6 and LX-2 were gifts from Prof. Qingsong Hu (USTC). HEK293T was purchased from ATCC. HCCLM3 was a gift from Prof. Hongyang Wang (SMMU).

Transfection was performed with Lipofectamine 3000 (Thermo). For lentiviral infection, HEK293T cells were transfected with psPAX2, pMD2.G, and PLKO.1/EZ-TetON-PLKO.1. The virus particles were then collected for infection. Cells were selected with puromycin (APExBIO). The inducible shRNA expression cells were cultured as reported and 50 ng ml^−1^ doxycycline (Sangon) was used to induce *SNRPD2*/*HDAC2* knockdown^57^.

### Plasmids

pcDNA3.1-HA and -FLAG were gifts from Prof. Qingsong Hu (USTC). pLKO.1-puro, psPAX2, and pMD2.G were gifts from Prof. Mian Wu (USTC). CMV-SB13 was kindly provided by Prof. Amaia Lujambio (ISMMS). pT3-EF1A-*MYC*-IRES-Luc (129775), pX330-*p53* (59910), and EZ-Tet-pLKO-Puro (85966) were purchased from Addgene.

*GCN5, PCAF, KAT5* (which encodes Tip60), *CREBBP*, *EP300* and *HDAC2* cDNA were amplified and cloned into the pcDNA3-FLAG vector. *SNRPD2* cDNA and its site-directed mutagenesis were constructed into pcDNA3.1-HA vector. The *BRCA1* minigene construct was generated by inserting the DNA fragment containing the human *BRCA1* genomic sequence from exon 8 to exon 10 into the pcDNA3.1 vector. Mutagenesis of minigene and *SNRPD2* constructs was performed using PCR and verified by sequencing. For generating CRISPR-Knockout murine HCC, sgRNAs were inserted into the PX330, replacing sg-*P53*. The sequences for sgRNAs, shRNAs used in this study are listed in Supplementary Table 1.

### Antibodies

The antibodies were as follows: anti-Ack rabbit antibody (9441s, 1:800 for Immunoblot), anti-HA mouse mAb (2367, 1:1000 for Immunoblot and 1:100 for immunofluorescence), anti-HA rabbit mAb (3724, 1:1000 for Immunoblot), anti-HDAC2 rabbit mAb (57156, 1:1000 for Immunoblot), anti-Poly/Mono-ADP Ribose rabbit mAb (83732, 1:1000 for Immunoblot), anti-p300 rabbit mAb (86377, 1:1000 for Immunoblot and 1:300 for immunofluorescence), anti-Flag rabbit mAb (14793, 1:1000 for Immunoblot), anti-CBP rabbit mAb (7389, 1:1000 for Immunoblot), anti-Vinculin rabbit mAb (13901, 1:1000 for Immunoblot), anti-BRCA1 rabbit mAb (9010, 1:1000 for Immunoblot and 1:100 for IHC), anti-β-Actin mouse mAb (3700, 1:1000 for Immunoblot), anti-PARP1 rabbit mAb (9532, 1:1000 for Immunoblot and 1:50 for IHC) and Rabbit mAb IgG Isotype Control (3900) are from Cell Signaling Technology. Anti-SmD2 antibody rabbit antibody (198296, 1:1000 for Immunoblot and I:200 for IHC) and anti-gamma H2A.X (phospho S139) rabbit antibody (81299, 1:1000 for Immunoblot, 1:500 for immunofluorescence, and I:200 for IHC) are from Abcam. Anti-FANCA rabbit antibody (11975-1-AP, 1:800 for Immunoblot and 1:50 for IHC) and anti-FANCD2 rabbit antibody (28619-1-AP, 1:800 for Immunoblot and 1:50 for IHC) are from Proteintech. Alexa Fluor 488 conjugated goat anti-mouse IgG (A11001), Alexa Fluor 488 conjugated goat anti-rabbit IgG (A11008). Alexa Fluor 555 conjugated goat anti-mouse IgG (A21422), Alexa Fluor 555 conjugated goat anti-rabbit IgG (A21428) and Alexa Fluor™ 555 Phalloidin (A34055) are from Thermo Fisher Scientific.

### Immunoprecipitation

For SmD2 acetylation analysis, cells were lysed in NETN lysis buffer (150 mM NaCl, 1 mM EDTA, 20 mM Tris pH 8, 0.5% Igepal CA-630) added with protease/phosphatase inhibitor (PPC1010, Sigma), 1 mM sodium butyrate (S1999, Selleck) and 2 μM TSA. For interaction analysis, cells were collected and lysed in lysis buffer (20 mM HEPES, pH 7.4, 150 mM NaCl, 1 mM EDTA, 0.1% Triton X-100) supplemented with a protease inhibitor cocktail. Cell lysates were clarified by centrifugation and incubated with magnetic beads (88802,88837 and A36797, Thermo) and or not anti-SmD2/Ac-K antibody at 4 °C with gentle rotation. After washing three times with lysis buffer, the beads were boiled and assessed by Immunoblot.

### Immunoblot

Protein lysates from cells were isolated using ice-cold RIPA buffer (Beyotime) supplemented with Protease and Phosphatase Inhibitor Cocktail (Sigma, #PPC1010). Lysates were incubated on ice for 20 min and clarified by centrifugation at 14,000 × *g* for 20 min at 4 °C. Proteins were then separated on an 8-20% Tris-Gly gel (Beyotime) and transferred to a PVDF membrane (Qiagen) for subsequent incubation with primary antibodies. Chemiluminescence signals were detected using ECL Western Blotting Substrate (Thermo) and a ChemiDoc imaging system (BioRad).

### LC-MS/MS

The immunoprecipitated products after elution by glycine (0.1 M, pH 2.0) and neutralization by HEPES were analyzed by liquid chromatography-tandem mass spectrometry. MS data were searched against the Swiss-Port database of Homo Sapiens. The list of proteins as putative interactors of SmD2 was in Supplementary Data 2.

### Immunohistochemistry

Tissue sections were deparaffinized and treated with Antigen Unmasking (Vector Labs). Then, they were preincubated with 10% goat serum and incubated with a primary antibody overnight at 4 °C. The tissue sections were then incubated with a biotinylated secondary antibody (Vector Labs) for one hour at room temperature and then with an ABC reagent (Vector Labs) for 30 min. The peroxidation reaction was conducted with a diaminobenzidine (DAB) kit (Vector Labs), and the slides were counterstained with hematoxylin (Sigma).

### Immunofluorescence

Cell monolayers and 3D spheres were first fixed with 4% paraformaldehyde for 10 min and then permeabilized with 0.2% Triton X-100 for 3 min. After blocking with PBS containing 0.05% Tween 20 and 1% bovine serum albumin (Sigma) for 1 h at room temperature, the fixed cells were incubated first with primary antibodies overnight at 4 °C and then with secondary antibodies for 1 h at room temperature. Finally, an antifade reagent with 4’6 diamidino-2-phenylindole (DAPI; Vector Labs) was used. All images were acquired using a confocal laser scanning microscope (Zeiss LSM 800).

### RNA extraction and semiquantitative/quantitative PCR

According to the manufacturer’s recommendations, total RNA was isolated with a GeneJET RNA Purification Kit (Thermo Fisher), and cDNA was synthesized with PrimeScript RT Master Mix (Takara). qPCR was performed with TB Green Premix Ex Tan II (Takara). Semiquantitative PCR was performed with Phanta Max Super-Fidelity DNA Polymerase (Vazyme). 18S RNA was utilized as an internal reference for U snRNA qPCR analysis, while β-Actin was used as an internal reference for mRNA. The sequences of primers used are listed in Supplementary Table 1.

### mRNA stability assay

The mRNA stability assay was carried out as described previously^58^. To measure mRNA half-life using qPCR, SmD2-deficient HCCLM3 cells infected with *UPF1* shRNA and shControl lentivirus were treated with 2.5 μg/ml Actinomycin D (A4448, Selleck) and collected at specified time intervals. *BRCA1* exclusion, *FANCD2* exclusion, and *FANCA* exclusion levels were determined through RT-PCR, as described previously. 18S RNA as an internal reference for qPCR.

### Drug treatment

Romidepsin (A8173), Belinostat (A4096), Vorinostat (A4084), Olaparib (A4154), Rucaparib (A8893), Niraparib (A3617), and Talazoparib (A4153) were purchased from APExBIO. Panobinostat (S1030), Trichostatin A (S1045), Nicotinamide (S1899), C646(S7152) and Cycloheximide (S7418) were purchased from Selleck. In the animal toxicity study, C57BL/6 mice were administered treatment starting at the age of 5 weeks, and the assay kits for aspartate aminotransferase (AST) (ab105135) and creatinine (ab65340) were purchased from Abcam.

### Inhibitor experiments

The cells were seeded in quadruplicates in 96-well plates at a density of 1000–3000 cells per well for the inhibitor experiments. Human HCC cell lines (HCCLM3, Huh7, and PLC/PRF/5) were treated with various inhibitors at different concentrations for 48–72 h. The cell viability was measured using the CCK-8 assay kit (Dojindo). To calculate the Combination Index (CI) for assessing drug interactions, we used CalcuSyn software (Biosoft). This method, based on the Chou-Talalay principle^42^, allows for the analysis of synergistic, additive, or antagonistic effects between drugs. After treating cells with both single agents and combinations at various concentrations, cell viability was measured. These data were inputted into CalcuSyn to compute the CI values, which indicate interaction types: CI < 1 suggests synergy, CI = 1 implies additivity, and CI > 1 indicates antagonism.

### Proliferation assays

For 3D culture, single cells were isolated, and 30,000 cells were mixed with 80 μl of ice-cold Matrigel (Corning) and seeded on 4 chambered cover glasses (Nunc/Thermo). The mixture was solidified by incubation at 37 °C for 15 min and then overlaid with 500 μl of medium. Cells were grown in the gel for 6‒8 days until spheres formed. For the colony formation assay, 1000–3000 cells per condition were plated in triplicate and cultured for 6~8 days, and the colony numbers were analyzed by Fiji.

### RNA-seq

RNA integrity was evaluated using an Agilent 2100 Bioanalyzer (Agilent Technologies). The samples with an RNA integrity number (RIN) ≥ 7 were used for subsequent analysis. According to the manufacturer’s instructions, libraries were constructed using a TruSeq Stranded mRNA LT Sample Prep Kit (Illumina). Then, these libraries were sequenced on an Illumina sequencing platform (HiSeqTM 2500 or Illumina HiSeq X Ten), and 125 bp/150 bp paired-end reads were generated. Raw data (raw reads) were processed using Trimmomatic^59^. Reads containing poly-Ns and low-quality reads were removed to obtain clean reads. Then, the clean reads were mapped to the reference genome using HISAT2^60^. The fragments per kilobase of transcript per million mapped reads (FPKM) value of each gene was calculated using cufflinks^61^, and the read count of each gene was obtained with HTseq^62^. DEGs were identified using the estimateSizeFactors and nbinomTest functions in the DESeq (2012). GO term and KEGG pathway enrichment analyses of the DEGs were performed using R based on the hypergeometric distribution^63^. Differential alternative splicing events were analyzed with rMATS software^64^ (version 4.0.1). The results of differential gene expression from RNA-seq and the alternative splicing outcomes derived from rMAT analysis based on RNA-seq data are provided in Supplementary Data 1.

### Gene set enrichment analysis

Gene Set Enrichment Analysis (GSEA) was performed using the software package (version 3.0) provided by the Broad Institute^65^. The gene expression data were preprocessed by filtering out genes with low expression levels and normalization. Gene sets from the Molecular Signatures Database (MSigDB) was used as the reference set^66^. The Enrichment Score (ES) was calculated by comparing the rank of the genes within the gene set to their ranks in the entire dataset. The significance of the ES was assessed by permutation analysis (*n* = 1000).

### Calculation of splice site strength, RNA binding energy and polypyrimidine tract analysis

Constitutive exons were obtained from HEXEvent^67^, while cassette exons were identified using RNA-seq data. Reference alternative exons, which are not regulated by SmD2, were determined by excluding exons affected by SmD2 based on data from HEXEvent. Splice site strengths for both types of exons were determined using maximum entropy models^68^. Additionally, the interaction binding energy between U1 snRNA and 5’ss was computed using the RNA-fold algorithm^69^, which is part of the ViennaRNA package v2.5.0. The analysis of poly-pyrimidine tracts was performed using the method developed by André Corvelo and colleagues^70^, available at https://github.com/omiics/PPT_finder10.

### Sequence logo analysis and RNA-seq read coverage plots

Read coverage sashimi plots were analyzed using the Integrative Genomics Viewer (IGV) from alignment/map (BAM) files in RNA-seq^71^. Sequence logo plots, summarizing the nucleotide consensus sequences for 5 splice sites, 3 splice sites and polypyrimidine tracts, were created by generating and illustrating position weight matrices using the ggseqlogo packages^72^.

### Animal

The wild-type C57BL/6 and nude mice were procured from Shanghai SLAC Laboratory Animal while NSG and C57BL/6-*MYC*^Cre/+^ mice were procured from Shanghai Model Organisms. All animals were bred at the First Affiliated Hospital of USTC and underwent a comprehensive examination before conducting the experiments to ensure their well-being and adaptation to the laboratory environment. Ethical guidelines were observed and approved by the Animal Ethics Committee of the First Affiliated Hospital of USTC for all animal experiments. Animals were humanely euthanized upon showing symptoms or when tumor volume reached a diameter of 2 cm or greater, in accordance with survival curve and end-stage analysis protocols. This practice was endorsed as a humane endpoint by the Animal Ethics Committee of the First Affiliated Hospital of the University of Science and Technology of China.

### Subcutaneous tumor

3–5 million cells per sample were injected subcutaneously into 5-week-old male nude mice or C57BL/6 for the mouse tumor model. Mice implanted with HCCLM3 or Hepa1-6 cells were euthanized after 4–5 weeks, and their tumors were isolated for subsequent analysis. For the creation of inducible shRNA cells, we adhered to the procedure described in the literature^73^. Cells infected with Tet-on shRNA virus underwent puro selection for one week to establish stable expression. These cells were then uniformly injected subcutaneously into mice, and tumor sizes were measured using calipers. The volume was calculated as Length × Width × Width × 0.5. Once tumors were established (typically around 4–6 days), mice were grouped into equal-volume treatment and doxycycline-induced groups. Starting from day 1 post-grouping, the doxycycline group received 50 mg/kg doxycycline orally every day to induce and maintain shRNA expression.

### Myc-driven HCC

The method of establishing the *H11-LSL-Myc* mouse liver cancer model was described in previous studies^43^. Firstly, Shanghai Model Organisms Center, Inc. developed the *H11LNL-Myc* knock-in mouse model through CRISPR/Cas9 system on the C57BL/6 mouse background. This was achieved by inserting the pCAG*-loxp-Neo-loxp-Myc-*polyA fragment into the well-defined Igs2 locus (the Hipp11 or H11 locus) via homologous recombination. *H11LNL-Myc* heterozygous mice were then bred with *Alb*-cre transgenic mice to generate *c-Myc*/*Alb-cre* double-positive mice for hepatocyte-specific overexpression of c-Myc, which activates c-Myc signaling and induces murine HCC. Tumor tissues were collected from HCC mice aged around two months and subcutaneously passaged on 4-week-old male C57BL/6 mice. After a month, the next generation of tumors were amplified and randomly divided into four groups to ensure that each group had an equal average tumor size before treatment.

### Spontaneous models of HCC by hydrodynamic tail vein injection

To better reproduce the role of *SNRPD2* in HCC, we used a previously reported model of spontaneous HCC tumorigenesis^22^. In brief, we prepared 13 μg of pT3-EF1a-*MYC*-IRES-luciferase, 10 μg of px330-sg-*p53*, 10 μg of px330-sg-*SNRPD2* (or sg-NonTarget) plasmids and a fourfold amount of CMV-SB13 (8.25 μg) dissolved in 2 ml of normal saline and injected a volume equivalent to 10% of the body weight of each mouse (male, 5 weeks old) via the tail vein over 3–5 s. The plasmids for tail vein injection were produced using a Qiagen Plasmid Midi Kit (Qiagen).

In vivo bioluminescence imaging was performed using an IVIS Spectrum system to quantify liver tumor burden. Mice were imaged 5 min after intraperitoneal injection with fresh D-luciferin (150 mg/kg) (GoldBio). The luciferase signal was quantified using Living Image software and then calculated by subtracting the background signal.

### PDX model

PDX model generation was as described previously^74^. In brief, the F0 of PDX specimens should be transported to the laboratory in tissue preservation solution (MACS) within 2 h at 4 °C under sterile conditions. Following this, the tissue samples should be minced and suspended in a mixture of 1:1 matrigel (Corning) and serum-free DMEM. The prepared suspension can then be injected into NSG mice (male, 6–8 weeks old) with an 18 G needle. Once the tumor has reached the desired size, fragments of the xenotransplants may be immediately implanted into NSG mice for further generations (F1…FN), cryopreserved for future passages, or drug treatment under experimental designs.

### Clinical patients

Label-free quantitative proteome analysis and Sm proteins expression analysis were performed on specimens from HCC patients undergoing surgery at the First Affiliated Hospital of USTC between June and December 2021. PDX models were established using the specimen procured from one HCC patient scheduled for surgical treatment in September 2022. Informed consent was acquired from all participants, and the study received approval from the Institutional Review Board. Two HCC tissue microarrays (TMAs) were used, with TMA1 containing 48 formalin-fixed, paraffin-embedded HCC tissues and TMA2 consisting of 58 HCC tumor and adjacent normal tissues. These samples were collected from patients who underwent curative resection at the Department of Hepatobiliary Surgery, First Affiliated Hospital of USTC. Ethical approval was granted (approval number 2022-KY293), and informed consent was obtained from all patients. Detailed information on the patients can be found in Supplementary Table 2.

### Label-free quantitative proteomic analysis

Protein Extraction: tissue samples were processed by grinding cells with liquid nitrogen, suspending them in lysis buffer (8 M urea, 1% protease inhibitor cocktail), sonicating thrice on ice, and centrifuging at 12,000 × *g* for 10 min at 4 °C. The supernatant was collected, and debris was discarded. LC-MS/MS Analysis: Tryptic peptides were eluted in solvent A (0.1% formic acid, 2% acetonitrile in water) and loaded onto a homemade reversed-phase analytical column (25-cm length, 75/100 μm i.d.). Peptides were separated using a gradient of solvent B (0.1% formic acid in acetonitrile) over 70 minutes, followed by 14 min and 3 min at different concentrations. The flow rate was maintained at 450 nL/min on a nanoElute UHPLC system (Bruker Daltonics). Mass Spectrometry Analysis: Peptides were analyzed using the timsTOF Pro (Bruker Daltonics) with a capillary source and an electrospray voltage of 1.60 kV. Precursors and fragments were analyzed at the TOF detector with an MS/MS scan range of 100–1700 m/z. The timsTOF Pro operated in PASEF mode, selecting precursors with charge states 0 to 5 for fragmentation and acquiring 10 PASEF-MS/MS scans per cycle. Dynamic exclusion was set to 30 s. Database Search: MS/MS data were processed using MaxQuant search engine (v.1.6.15.0), searching tandem mass spectra against the human SwissProt database concatenated with the reverse decoy database. Trypsin/P was specified as the cleavage enzyme, allowing up to 2 missing cleavages. Mass tolerances for precursor and fragment ions were set to 20 ppm (first search), 5 ppm (main search), and 0.02 Da, respectively.

### TCGA analysis

As for the TCGA datasets, the data were downloaded from UCSC XENA and processed via the Toil workflow for uniform handling of TCGA and GTEx RNAseq data. We extracted RNAseq data of LIHC (Liver Hepatocellular Carcinoma) from TCGA and corresponding normal tissue data from GTEx. Differential comparisons were conducted using the limma [3.46.0] R package. For the survival analysis, we utilized GEPIA2 (Gene Expression Profiling Interactive Analysis 2) (http://gepia2.cancer-pku.cn/), with the grouping for Kaplan–Meier survival analysis based on a 50% cutoff of target RNA expression values^75^. We used the limma (v.3.46.0) R package to compare differences and ggplot2 (v.3.3.3) for visualization.

### Statistics & reproducibility

The sample size for the animals was not determined using a statistical method. Instead, the selection was guided by our familiarity with the experimental models commonly employed in cell biology and animal research. All data shown were obtained from at least three biological independent experiments with similar results. Statistical analyses were conducted using Student’s *t* test, the binomial distribution test, the Mann–Whitney U test, or two-way Analysis of Variance (ANOVA), employing either GraphPad Prism [10.1.0] or R software [4.3.2]. The Kaplan–Meier method (log-rank test) was utilized to assess cumulative survival rates.

### Reporting summary

Further information on research design is available in the Nature Portfolio Reporting Summary linked to this article.

### Supplementary information

Supplementary Information

Description of Additional Supplementary Files

Supplementary Data 1

Supplementary Data 2

Reporting Summary

### Source data

Source Data

## Data Availability

RNA-seq data generated of this study have been deposited in the Genome Sequence Archive Human [HRA005004]. The Label-Free Quantitative Proteomics MS data are being deposited in PRIDE [PXD043544]. The MS data of immunoprecipitation against SmD2 are being deposited in PRIDE [PXD043535]. The dataset derived from this resource that supports the findings of this study is available at GEPIA2. Regarding the TCGA datasets, these were obtained from UCSC XENA, available at https://xenabrowser.net/datapages/. The remaining data are available within the Article, Supplementary Information or Source Data file. Source data are provided with this paper.
