# Supplementary material for: Acetylation-dependent regulation of core spliceosome modulates hepatocellular carcinoma cassette exons and sensitivity to PARP inhibitors
Source: Nat Commun. 2024 Jun 18;15:5209. doi: 10.1038/s41467-024-49573-7 (PMC11189467; doi:10.1038/s41467-024-49573-7)
Supplement: Supplementary file 1 — Supplementary Information [file 41467_2024_49573_MOESM1_ESM.pdf]

Supplementary information

Acetylation-Dependent Regulation of Core Spliceosome Modulates Hepatocellular Carcinoma Cassette Exons and Sensitivity to PARP Inhibitors

Linmao Sun[1,2,3,6]; Yufeng Liu[1,2,3,6]; Xinyu Guo[2,6]; Tianming Cui[1,2,3,6]; Chenghui Wu[1,2,6]; Jie Tao[2]; Cheng Cheng[1,2]; Qi Chu[1,2]; Changyong Ji[1,2]; Xianying Li[1,2]; Hongrui Guo[1,2,3]; Shuhang Liang[2,4]; Huanran Zhou[2,5]; Shuo Zhou[1,2]; Kun Ma[1,2,3]; Ning Zhang[2]; Jiabei Wang[1,2,3,*]; Yao Liu[1,2,3,*]; Lianxin Liu[1,2,3,*]

[1] Department of Hepatobiliary Surgery, Centre for Leading Medicine and Advanced Technologies of IHM, The First Affiliated Hospital of USTC, Division of Life Sciences and Medicine, University of Science and Technology of China, Hefei, Anhui, 230001, China.
[2] Anhui Province Key Laboratory of Hepatopancreatobiliary Surgery, Hefei, Anhui, 230001, China.
[3] Anhui Provincial Clinical Research Center for Hepatobiliary Diseases, Hefei, Anhui, 230001, China.
[4] Department of Gastrointestinal Surgery, The First Affiliated Hospital of USTC, Division of Life Sciences and Medicine, University of Science and Technology of China, Hefei 230001, China.
[5] Department of Endocrinology, The First Affiliated Hospital of USTC, Division of Life Sciences and Medicine, University of Science and Technology of China, Hefei 230001, China.
[6] These authors contributed equally.
[*]Correspondence: jbwang16@ustc.edu.cn (J.W.), liuyao66@ustc.edu.cn (L.Y.), liulx@ustc.edu.cn (L.L.).

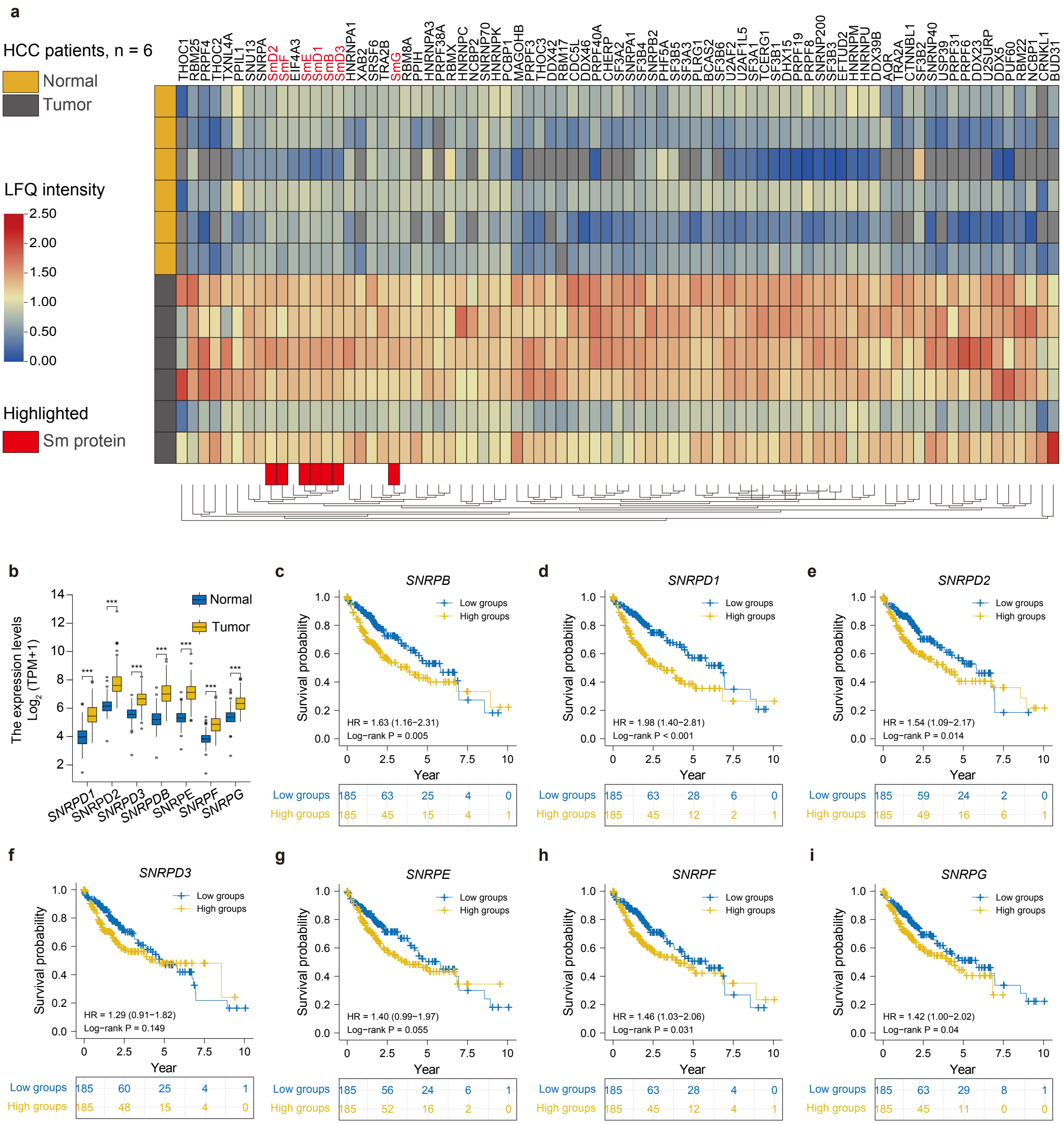

Supplementary Fig. 1 | Comparative analysis of Sm proteins expression levels in human HCC tissues.
a, Hierarchical clustering and heat-map analysis of differential LFQ intensity between tumor tissues and normal tissues from quantitative Proteome. Rows and columns represent samples and LFQ intensity, respectively, and 80 high-expression spliceosome proteins are shown.
b, Expression levels of 7 Sm proteins in human hepatocellular carcinoma (HCC) tissues (n=374) compared to normal liver tissues (n=50) from the TCGA-LIHC dataset. The center line in each box represents the median, the edges of the boxes represent the first (25th percentile) and third (75th percentile) quartiles, and the whiskers extend to the minimum and maximum values. Statistical significance was evaluated using Welch's t-test. ***, P < 0.001.
c-i, Kaplan-Meier survival curves of HCC patients stratified by SNRPB (**c**), SNRPD1 (**d**), SNRPD2 (**e**), SNRPD3 (**f**), SNRPE (**g**), SNRPF (**h**), and SNRPG (**i**) expression levels from the TCGA LIHC database reveal statistically significant differences in overall survival. Log-rank Mantel-Cox test.

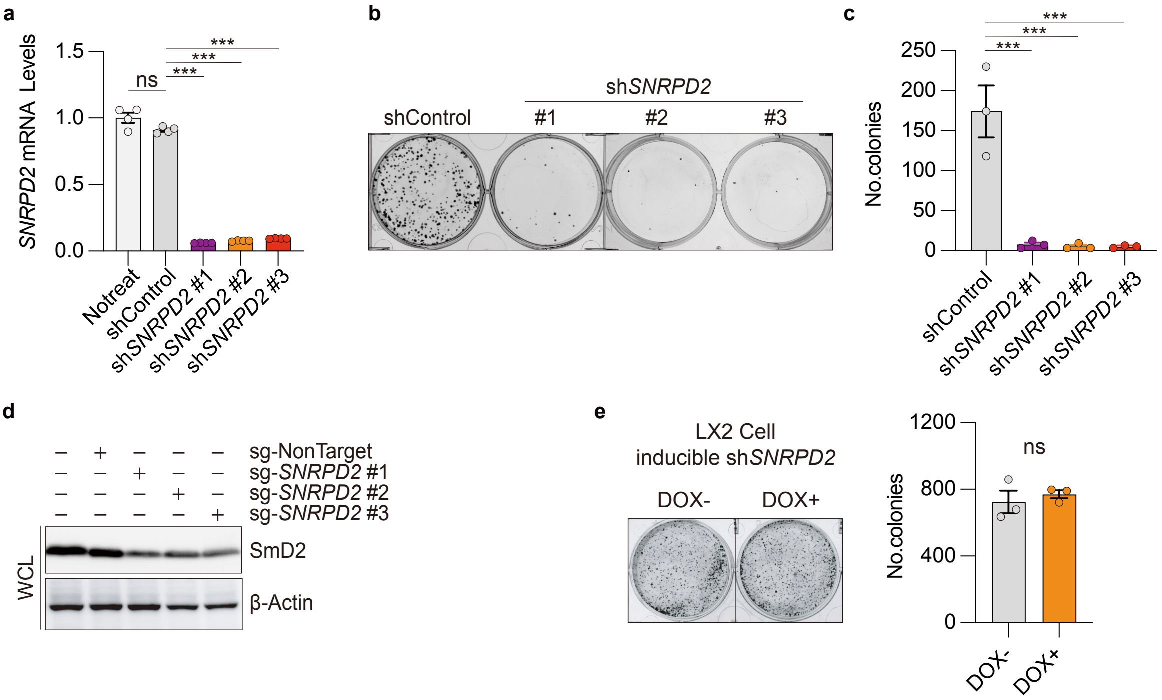

Supplementary Fig. 2 | Effects of sh*SNRPD2* and sg*SNRPD2.*

a, qPCR assays showing the efficiency of sh*SNRPD2* sequences in HCCLM3 cells, presented as mean values ± SD (n=4 biologically independent samples).

b, Representative images of colony-formation assays of HCCLM3 cells in three shRNAs against *SNRPD2*.

c, Number of colonies from B, presented as mean values ± SD (n=3 biologically independent experiments).

d, Immunoblot analysis of the whole-cell lysates (WCL) from Hepa1-6 cells transduced with PX330-sgNonTarget or PX330-sg*SN-RPD2* plasmid. The experiment was repeated three times with similar results.

e, Colony-formation assays to compare the effects of uninduced versus doxycycline-induced sh*SNRPD2* expression on LX-2 cells, presented as mean values ± SD (n=3 biologically independent experiments).

Statistical significance was determined using two-sided t-tests, ***$P < 0.001$.

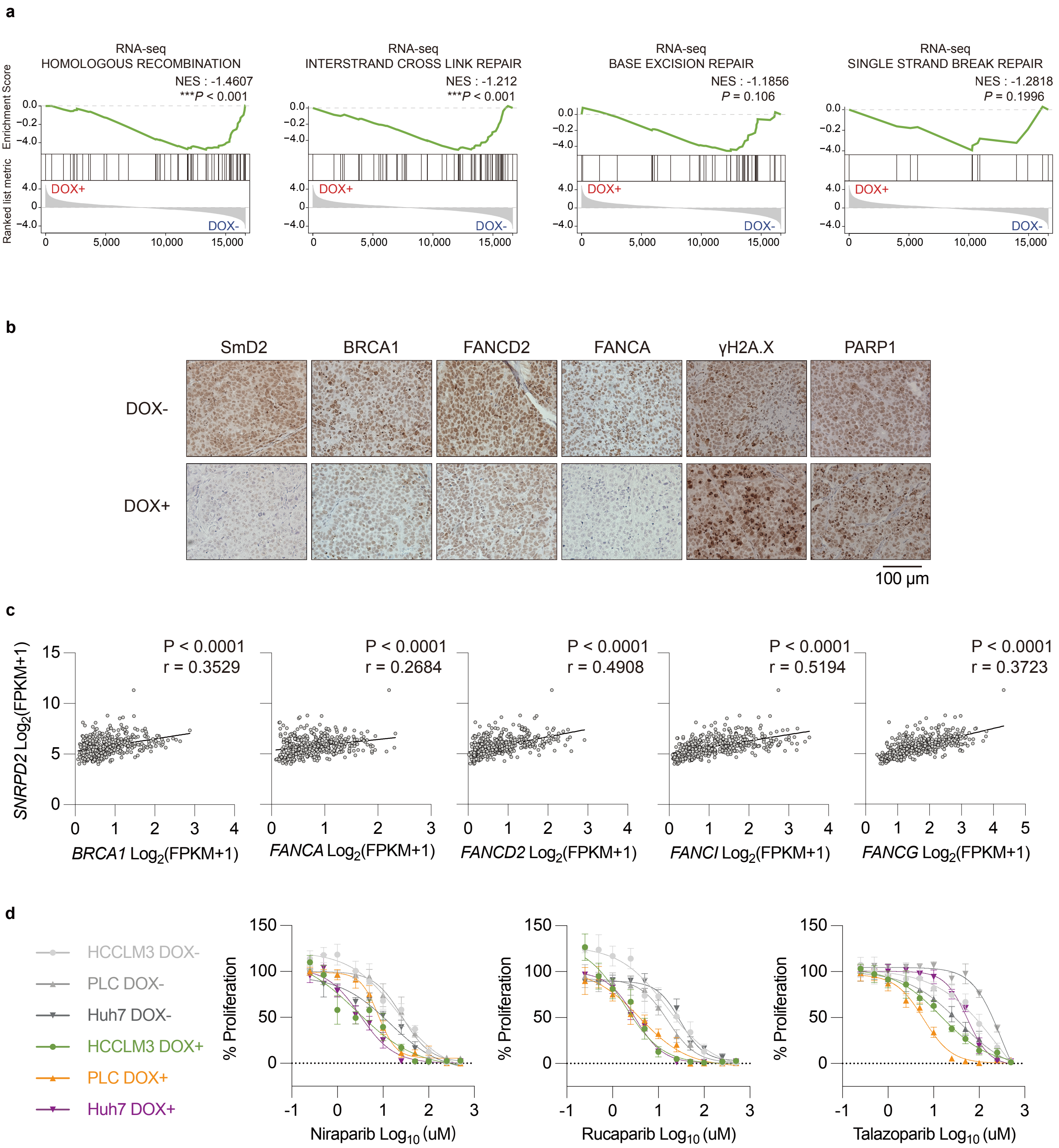

Supplementary Fig. 3 | Human relevance between SmD2 and DNA repair genes in HCC.
a, Gene set enrichment analysis (GSEA) plots displaying the relative expression of genes involved in homologous recombination, interstrand cross-link repair, base excision repair, and single-strand break repair pathways in HCC cell lines treated with doxycycline (DOX+ to induce sh*SNRPD2* expression) versus untreated (DOX-). Notable enrichment scores (NES) and p-values indicate the significance of the gene sets.
b, Immunohistochemical analysis of SmD2, BRCA1, FANCA, FANCD2, γH2A.X, and PARP1 in shSmD2-inducible HCCLM3 xenografts. The experiment was repeated three times with similar results.
c, Scatter plots showing the correlation between the expression of *SNRPD2* and DNA repair genes *BRCA1*, *FANCA*, *FANCD2*, *FANCI*, and *FANCG* in the TCGA LIHC dataset (n = 374). Pearson correlation coefficients (r) and p-values are provided, demonstrating significant correlations.
d, Dose-response curves of Niraparib, Rucaparib, and Talazoparib on shSmD2-inducible HCC cell lines over 72 hours (n = 4 biologically independent samples).

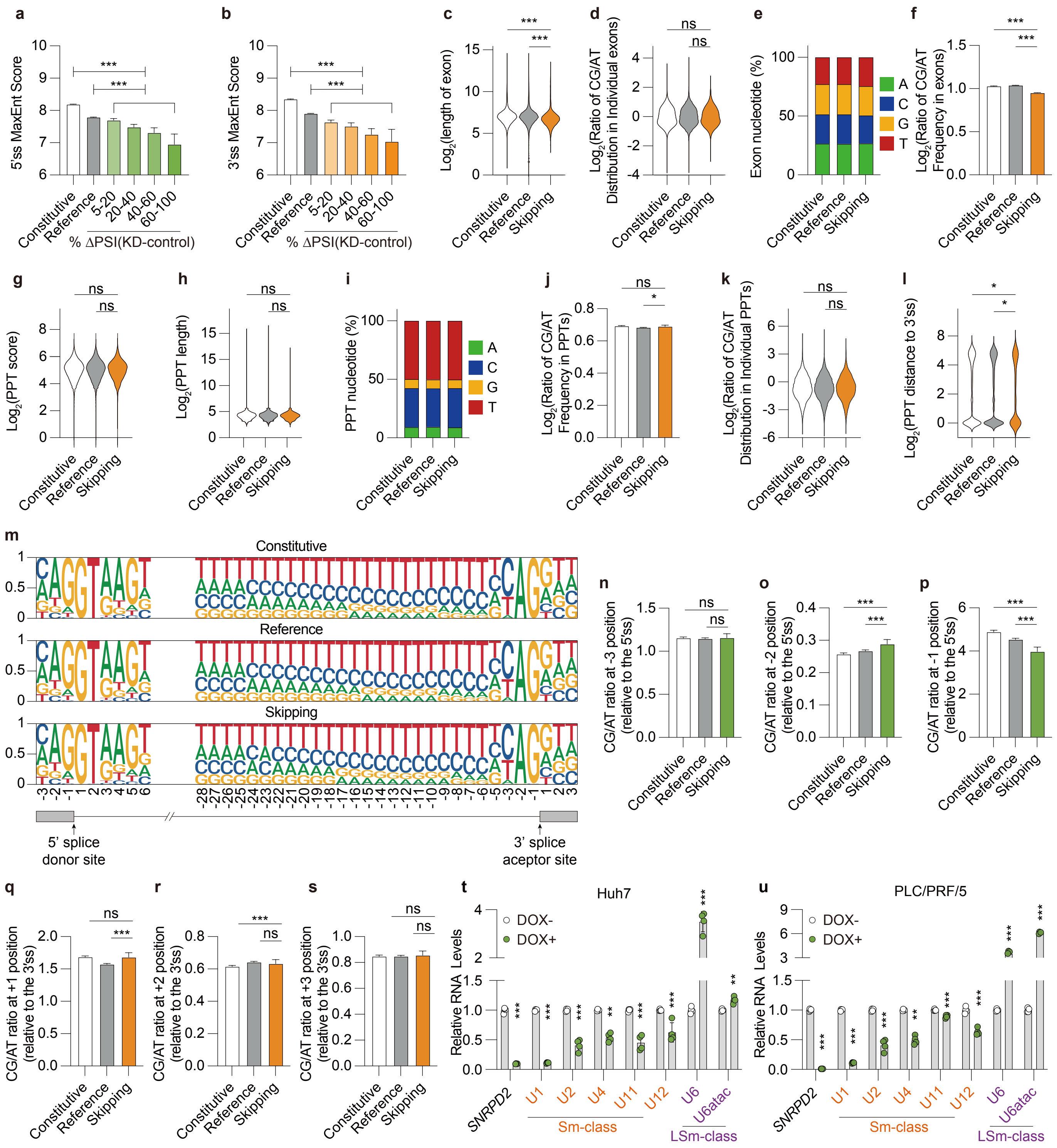

Supplementary Fig. 4 | Characteristics of SmD2-regulated exons.

a and b, 5′ss (donor sites) MaxEnt score (**a**) and 3′ss (acceptor sites) MaxEnt score (**b**) for skipping exons (influenced by SmD2 knockdown) compared to reference alternative (not regulated upon SmD2) and constitutive exons. PSI (Percent-Spliced-In), fraction of spliced mRNA. Error bars represent ±1 SEM. P value was estimated with two-sided t-test.

c, Lengths of exons for constitutive, reference, and skipping groups. one-way ANOVA

d, GC:AT nucleotides within individual exons, comparing constitutive, reference, and skipping groups. one-way ANOVA.

e, Composition percentages of exon nucleotides, categorized by A, C, G, and T.

f, GC:AT nucleotide frequency in exons. Error bars represent ±1 SD. estimated by bootstrapping (10,00 iterations). P value was estimated with a two-sided binomial proportion test.

g, Distribution of polypyrimidine tract (PPT) scores among constitutive, reference, and skipping exons. one-way ANOVA.

h, PPT lengths in constitutive, reference, and skipping exons. one-way ANOVA.

i, Proportional composition of nucleotides within PPTs.

j, Ratio of GC:AT Nucleotide Frequencies in PPTs. Error bars represent ±1 SD. estimated by bootstrapping (10,00 iterations). P value was estimated with a two-sided binomial proportion test.

k, Ratio of GC:AT nucleotides in individual PPTs across constitutive, reference, and skipping exons. one-way ANOVA.

l, PPT distance to 3′ss in exons. one-way ANOVA.

m, Sequence logo plots of the 5′ss, 3′ss and PPTs of constitutive, reference, and skipping exons.

n-s, GC:AT ratio at positions -3 (**n**), -2 (**o**), -1 (**p**) relative to the 5′ss, and at positions +1 (**q**), +2 (**r**), +3 (**s**) relative to the 3′ss. Error bars represent ±1 SD. estimated by bootstrapping (10,000 iterations). two-sided Mann–Whitney U test.

t and u, qPCR examining the changes of spliceosomal snRNAs in Huh7 (**t**) or PLC/PRF/5 (**u**) cells after doxycycline (DOX)-induced SmD2 knockdown (n=4 biologically independent samples).Mean = +/-SD, two-sided t-test.

*P < 0.05, **P < 0.01, ***P < 0.001.

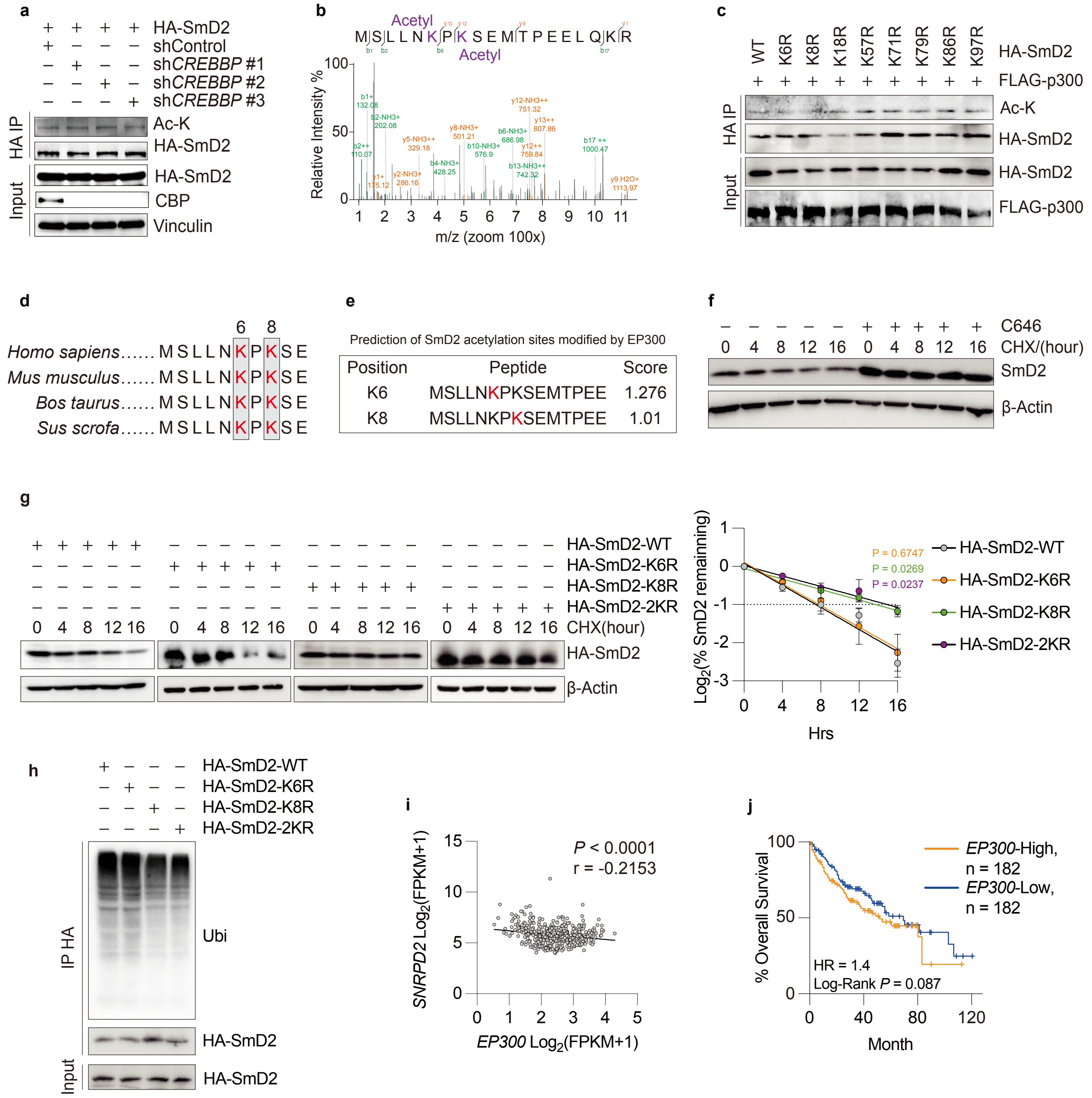

Supplementary Fig. 5 | The acetylation process of SmD2 impacts its stability.

a, Immunoblot analysis of input and anti-HA immunoprecipitates (IPs) derived from HEK293T cells transduced with HA-SmD2 and shRNAs against *CREBBP* . The experiment was repeated three times with similar results.
b, The SmD2 proteins from Fig. 5a were analyzed by mass spectrometry.
c, Immunoblot analysis of input and anti-HA IPs derived from HEK293T cells transfected with FLAG–p300 and HA-tagged SmD2 or K (lysine) to R (arginine) mutants. The experiment was repeated three times with similar results.
d, Alignment of SmD2 amino acid sequence from various species. Grey shading indicates the conserved Lys 6 and 8.
e, Predicted lysine acetylation sites by the GPS 2.0 (Group-based Prediction System, Ver 2.0) for KAT-specific Acetylation Site Prediction (ASEB) analysis.
f, Expression levels of SmD2 from HCCLM3 treated with the p300 inhibitor (20μM c646) overnight and cycloheximide (CHX,10μM). The experiment was repeated three times with similar results.
g, HCCLM3 cells stably expressing HA-tagged wild-type (WT) SmD2 or SmD2 mutants were treated with CHX, and the SmD2 protein levels were quantified (n=3 independent experiments).The *P*-value was calculated in comparison to the wild-type (WT) control group.
h, Immunoblot analysis of the input and anti-HA IPs obtained from HEK293T cells transfected with HA-tagged WT SmD2 or the SmD2 mutants. The experiment was repeated three times with similar results.
i, Positive correlations of *SNRPD2* mRNA levels with *EP300* mRNA levels in The Cancer Genome Atlas (TCGA) Liver Hepatocellular Carcinoma (LIHC) dataset. n = 424, 374 of which were tumor samples and 50 adjacent normal tissues. r, Pearson's correlation coefficient.
j, Kaplan-Meier survival analysis of HCC patients from TCGA LIHC dataset, stratified by *EP300* mRNA expression levels. Patients are categorized into two cohorts: those with EP300 expression above the median and those with expression below the median.

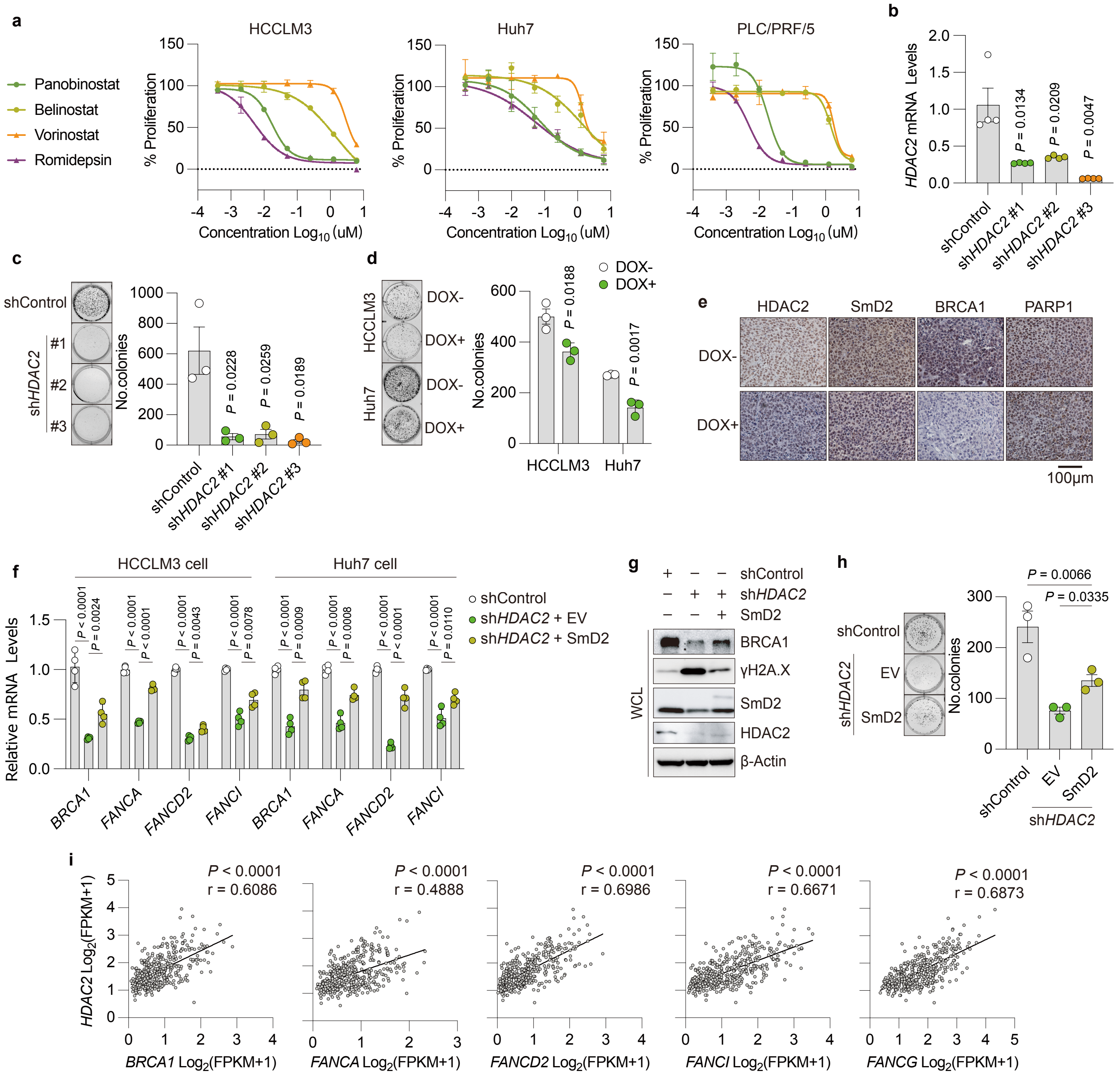

Supplementary Fig. 6 | HDAC2 modulate DNA damage response in HCC via SmD2 acetylation.

a, Dose-response curves of four FDA-approved HDAC inhibitors (Panobinostat, Belinostat, Vorinostat, and Romidepsin) over 48 hours in three HCC cell lines (n=4 biologically independent samples).

b, qPCR assays showing the efficiency of sh*HDAC2* sequences in HCCLM3 cells (n=4 biologically independent samples).

c, Colony-formation assays to assess the impact of three shRNAs against *HDAC2* on HCCLM3 cells (n=3 biologically independent experiments).

d, Colony-formation assays to compare the effects of uninduced or doxycycline-induced sh*HDAC2* expression on HCCLM3 and Huh7 cells (n=3 biologically independent experiments).

e, Immunohistochemical analysis of SmD2, BRCA1 and PARP1 in sh*HDAC2*-inducible HCCLM3 xenografts. Mice were treated (+) or not treated (−) with doxycycline (DOX) to induce *HDAC2* knockdown. The experiment was repeated three times with similar results.

f, qPCR assays demonstrating SmD2 restoration consequently rescue downregulated DNA repair genes in HCCLM3 and Huh7 cells silenced by *HDAC2* (n=4 biologically independent samples).

g, Immunoblot assays demonstrating the upregulation of BRCA1 expression and attenuation of DNA damage accumulation (γH2A.X) by SmD2 restoration in HCCLM3 silenced by *HDAC2*. The experiment was repeated three times with similar results.

h, Colony-formation assays displaying SmD2 restoration rescued the growth of HCCLM3 cells silenced by *HDAC2* (n=3 biologically independent experiments).

i, Positive Correlations between *HDAC2* mRNA expression and mRNA expression of *BRCA1*, *FANCA*, *FANCD2*, *FANCI*, and *FANCG* in the TCGA LIHC dataset. n = 424, 374 of which were tumor samples and 50 adjacent normal tissues. r, Pearson's correlation coefficient.

Data are presented as mean values ± SEM. Statistical significance was determined using two-sided t-tests.

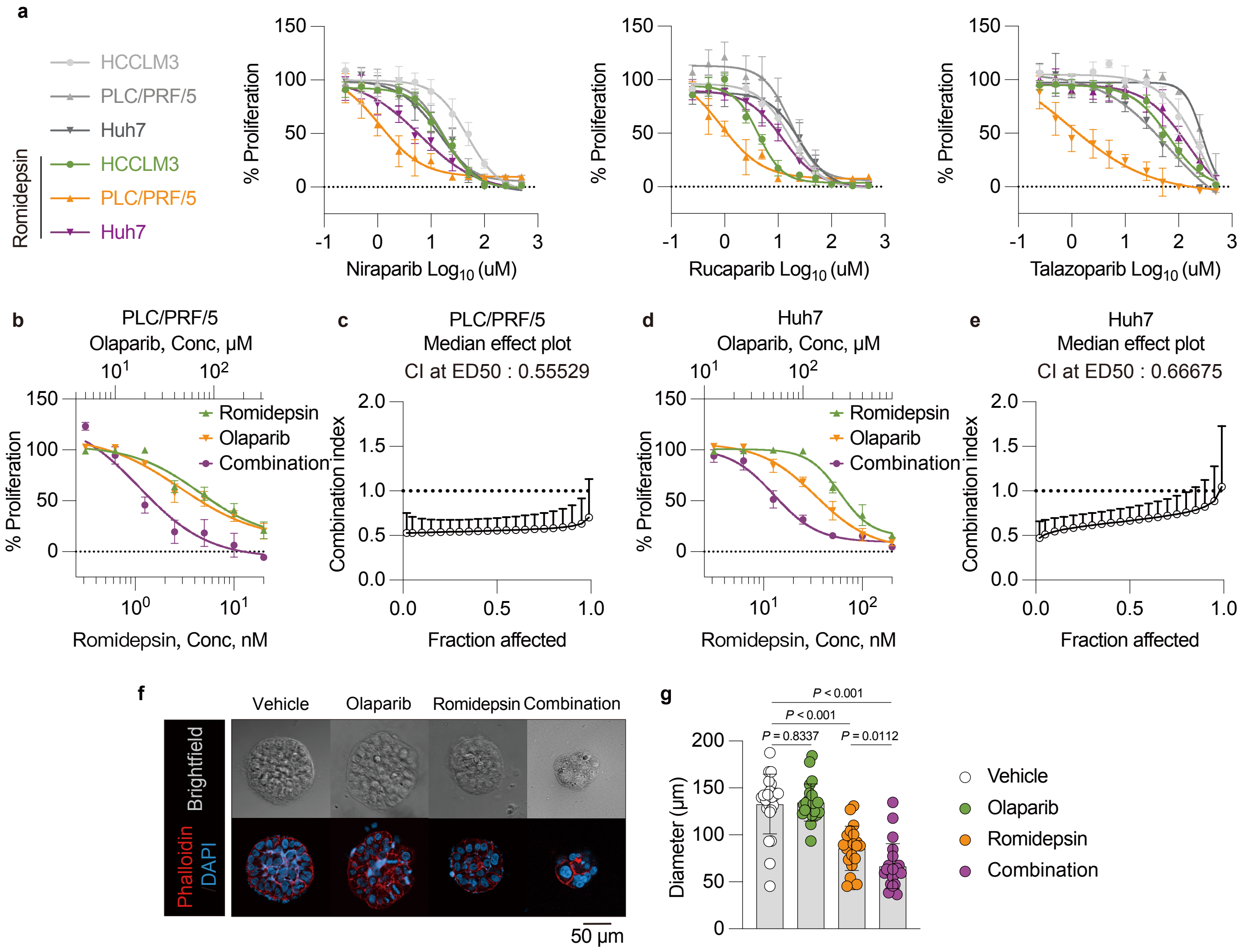

Supplementary Fig. 7 | Rational Combination Strategies with PARP Inhibitors and Romidepsin.

a, Dose-response curves of Niraparib, Rucaparib, and Talazoparib on HCC cell lines treated with Romidepsin (IC20). Mean values ± SD, n = 4 biologically independent samples.

b, Synergistic dose-response curves for PLC/PRF/5 cells treated with varying concentrations of Olaparib and Romidepsin (n=4 biologically independent samples).

c, Combination index (CI) versus fraction affected for Olaparib and Romidepsin in PLC/PRF/5 cells.

d, Dose-response as in (**b**) for Huh7 cells (n=4 biologically independent samples).

e, Combination index as in (**c**) for Huh7 cells.

f, Representative 3D culture plot of transfected inducible shSmD2 HCCLM3 cells after Romidepsin and Olaparib treatment.

g, Diameter of the cell spheres counted from (**f**). n=22 biologically independent samples. Mean values ± SEM. Two-sided t-tests were used to determine statistical significance.

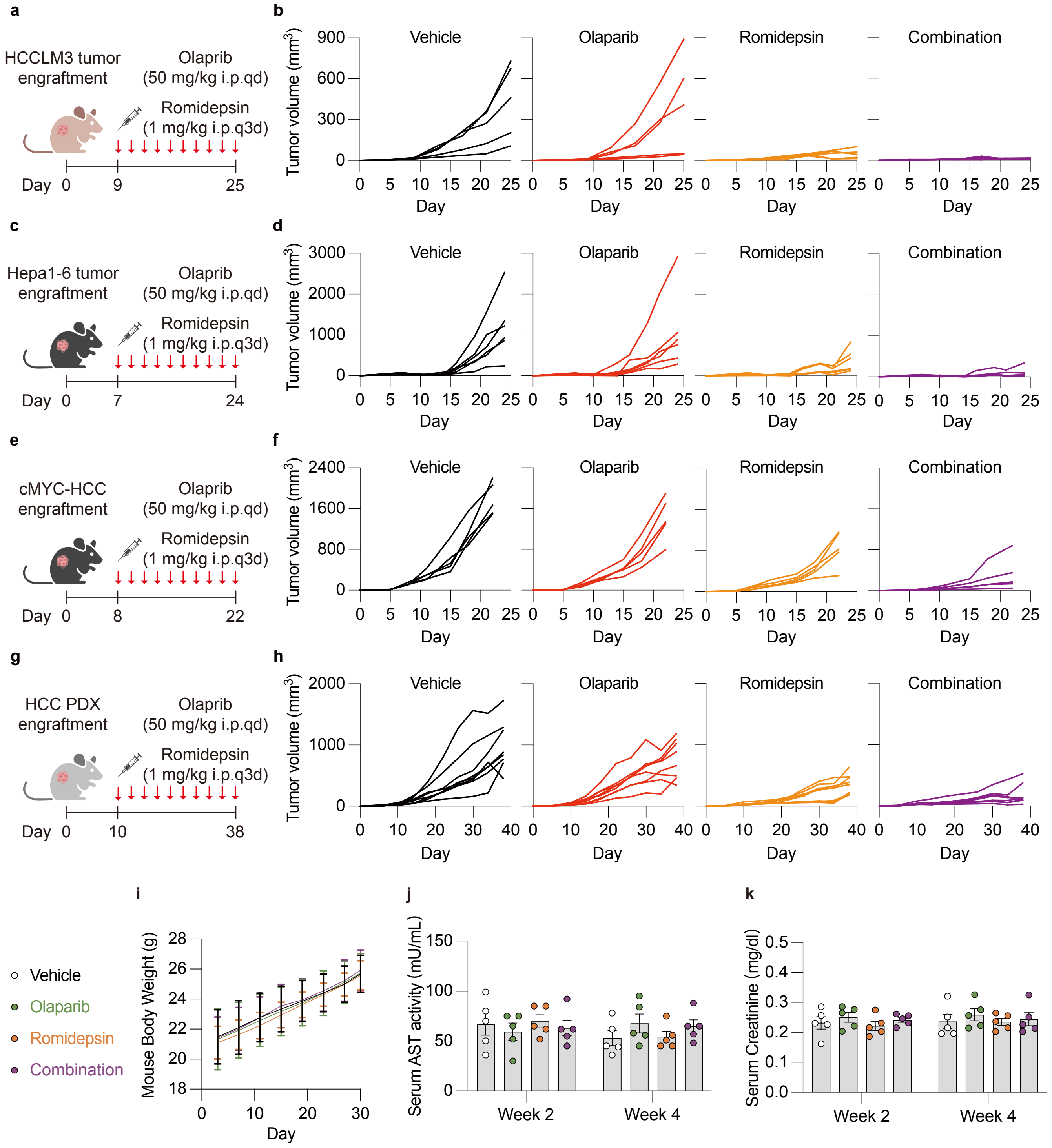

Supplementary Fig. 8 | Evaluation of Olaparib and Romidepsin Combination Therapy in Various HCC Models.
a, Treatment schema of HCCLM3 tumor-bearing nude mice (created with BioRender.com).
b, Individual tumor growth curves for vehicle, Olaparib, Romidepsin, or both. n = 5 per group; tumors engrafted on bilateral flanks.
c, Treatment schema of Hepa1-6 tumor-bearing C57BL/6 mice (created with BioRender.com).
d, As (**a**), but for Hepa1-6 tumor-bearing C57/BL6 mice (n = 6/group).
e, Treatment schema of c-MYC-driven-HCC tumor-bearing C57BL/6 mice (created with BioRender.com).
f, As (**a**), but for cMYC-driven HCC tumor-bearing C57/BL6 mice (n = 5/group).
g, Treatment schema of HCC PDX NSG mice (created with BioRender.com).
h, As (**a**), but for HCC PDX tumor-bearing NSG mice (n = 8/group).
i, Temporal variation in body weight of 6-week-old C57BL/6 mice subjected to various treatments (n=5/group).
j and k, Evaluation of serum aspartate aminotransferase (AST) levels (**q**) and serum creatinine concentrations (**r**) in mice at 2 and 4 weeks following treatment (n=5/group).
Figures 8**a**, 8**c**, 8**e**, and 8**g** created with BioRender.com, released under a Creative Commons Attribution-NonCommercial-NoDerivs 4.0 International license.

Supplementary Table 1 . Sequences of primers, shRNAs, sgRNAs.		
Usage	Primer name	Sequence (5'-3')
RT-qPCR primers	ACTB-F	CACCATTGGCAATGAGCGGTTC
	ACTB-R	AGGTCTTTGCGGATGTCCACGT
	SmB-F	TTGGCACCTTCAAGGCTTTTGAC
	SmB-R	AGACCGAGGACTCGCTTCTCTT
	SmD1-F	AGACAGTTTACCTCTGGATACACT
	SmD1-R	TCTTCCTCTGCCTCTTCCTGCA
	SmD2-F	CAAGTGCTCATCAACTGCCGCA
	SmD2-R	GCGGTCTTTGTTGACTGGCTTG
	SmD3-F	GGAAGCTCATTGAAGCAGAGGAC
	SmD3-R	CAGAAAGCGGATTTTGCTGCCAC
	SmE-F	CGGATTCAGGTGTGGCTCTATG
	SmE-R	CTCTTCTGCATCATCTAATACAAGG
	SmF-F	GGGAATGGAGTACAAGGGCTATC
	SmF-R	AAAACTTCACCCAGATGTCCAGAC
	SmG-F	GAATGTGTGGAGATGGCGACTAG
	SmG-R	GGGTTTCTCTGCTGAACAGCCA
	BRCA1-F	CTGAAGACTGCTCAGGGCTATC
	BRCA1-R	AGGGTAGCTGTTAGAAGGCTGG
	BARD1-F	TGCAGCCAAGAATGGGCATGTG
	BARD1-R	CTTCTCTGGTAGCAGCAATAGCG
	FANCA-F	CAGAACCCAACTCTGCTGAGGA
	FANCA-R	ATCACTGCCACCTGTGCCGATA
	FANCC-F	TGCCTGAGAAGGATGGAGTGCT
	FANCC-R	CCGTCAGTTTCCAGCAGCACAA
	FANCI-F	GCAAGCTGATGTTCGACTCATGC
	FANCI-R	AGGCAGCAGATCAGGTTTTGGC
	FANCD2-F	TTCCAGGATGCCTTCGTAGTGG
	FANCD2-R	GCAGGAGGTTTATGGCAATCCC
	FANCG-F	GAGAGTCTGGAGCTGCTAGTTG
	FANCG-R	TGTGCTTGGTCTGGCTCTGAGT
	FANCL-F	GGAGTGCAACAGCACGCAGAAT
	FANCL-R	CTGCTCAGCTTAATTCCCAGGG
	U1 snRNA-F	GATACCATGATCACGAAGGTGGTT
	U1 snRNA-R	CACAAATTATGCAGTCGAGTTTCC
	U2 snRNA-F	TTTGGCTAAGATCAAGTGTAGTATCTGTTC
	U2 snRNA-R	AATCCATTTAATATATTGTCCTCGGATAGA
	U4 snRNA-F	GCGCGATTATTGCTAATTGAAA
	U4 snRNA-R	AAAAATTGCCAATGCCGACTA
	U11 snRNA-F	GTGCGGAATCGACATCAAGAG
	U11 snRNA-R	CGCCGGGACCAACGAT
	U12 snRNA-F	AACTTATGAGTAAGGAAAATAACGATTCG
	U12 snRNA-R	CGACCTTTACCCGCTCAAAA
	U6 snRNA-F	GCTTCGGCAGCACATATACTAAAAT
	U6 snRNA-R	ACGAATTTGCGTGTCATCCTT
	U6 atac snRNA-F	AGGTTAGCACTCCCCTTGACAA
	U6 atac snRNA-R	TGGCAATGCCTTAACCGTATG
	RPS18 Human-F	GCTTGTTGTCCAGACCATTGGC
	RPS18 Human-R	GCAGAATCCACGCCAGTACAAG
	HDAC2-F	CTCATGCACCTGGTGTCCAGAT
	HDAC2-R	GCTATCCGCTTGTCTGATGCTC
Semi-quantitative RT-PCR primers	GAPDH-F	GCAAATTCCATGGCACCGTC
	GAPDH-G	TCGCCCCACTTGATTTTGG
	BRCA1-F	CCTTCCTTGCAGGAAACCAGTC
	BRCA1-R	ATGGCTCCACATGCAAGTTTGA
	FANCA-F	CTCTGCTCTGGTGTGGTGG
	FANCA-R	CAGGTCTGTGGTGCTATTTGAGG
	FANCD2-F	GGCCAGCTAAACAAGGAGTTC
	FANCD2-R	GATTCTCAGCAGCTAAACACTGAAAATAG
	FANCG-F	ATAGTCTGCAAGGGCTCCC
	FANCG-R	AGCTCCTGGAGACCTTGGC
	FANCI-F	CCATAAGTAGTTTCAGTAAGAATAGGTTTGAGG
	FANCI-R	CTTATCTGTGACATCCAGAGCTCTGAG
Semi-quantitative RT-PCR primers for U1 snRNA mutations	Exon9-BRCA1-F	ATTGAATTGGGATCTGATTC
	Exon9-BRCA1-R	ACATTCATTCTGTCTTTAG
shRNAs for candidate proteins knock down.The TetON system was constructed using SmD2 #2 and HDAC #1.	Scramble	AACGTACGCGGAATACTTCGA
	SmD2 #1	CATCAACTGCCGCAACAATAA
	SmD2 #2	GCTCACACAGTCAGTCAAGAA
	SmD2 #3	CGATAGGCACTGCAACATGGT
	p300 #1	CCCGGTGAACTCTCCTATAAT
	p300 #2	ATACTCAGCCGGAGGATATTT
	p300 #3	TAACCAATGGTGGTGATATTA
	HDAC2 #1	GACGGTATCATTCCATAAATA
	HDAC2 #2	CAGACTGATATGGCTGTTAAT
	HDAC2 #3	CAGTCAAAGGTCATGCTAAAT
	UPF1 #1	AGATATGCCTGCGGTACAAAG
	UPF1 #2	TTACCTTGGTGACGAGTTTAA
	CREBBP #1	ATCGCCACGTCCCTTAGTAAC
	CREBBP #2	CGTTTACCATGAGATCCTTAT
	CREBBP #3	GGGATGAATATTATCACTTAT
sgRNAs for Hydrodynamic tail vein injection	NonTarget	GCGGGCAGAACGACCCTGAC
	SmD2 #1	GCGCGGGGACTCGGTCATCG
	SmD2 #2	GAAATCCAAGCCTGTCAACA
	SmD2 #3	AACGGGCATGAGACTCTCCG

Supplementary Table 2: Detailed Patient Data for Proteomic Analysis								
Patients	Gender	Age	Pathological Grade	TNM Stage	MVI	Treatment history		
						Surgical treatment	Immunotherapy	Targeted therapy
P189	Male	>60	Ⅱ	Ⅲb(T4)	M1	Yes	No	No
P140	Female	45~60	Ⅱ~Ⅲ	Ⅲb(T4)	M2	Yes	Sintilimab	No
P118	Male	>60	Ⅲ~IV	Ⅲb(T4)	M2	Yes	No	Lenvatinib
P112	Male	45~60	Ⅲ	Ⅲb(T4)	M2	Yes	Camrelizumab	Lenvatinib
P207	Male	45~60	Ⅲ	Ⅲb(T4)	M2	Yes	No	No
P208	Male	45~60	Ⅲ	Ⅲb(T4)	M2	Yes	No	No
