# Supplementary material for: Acetylation-dependent regulation of core spliceosome modulates hepatocellular carcinoma cassette exons and sensitivity to PARP inhibitors
Source: Nat Commun. 2024 Jun 18;15:5209. doi: 10.1038/s41467-024-49573-7 (PMC11189467; doi:10.1038/s41467-024-49573-7)
Supplement: Supplementary file 2 — Description of Additional Supplementary Files [file 41467_2024_49573_MOESM2_ESM.pdf]

Description of Additional Supplementary Files

Supplementary Data 1: Differentially expressed genes after doxycycline-induced SmD2 Knockdown from RNAseq (two-sided wald test). And splicing Events Affected by SmD2 Knockdown in rMAT Analysis (two-sided likelihood ratio tests): Alternative 3' Splice Site (A3SS), Alternative 5' Splice Site (A5SS), Alternative 5' Splice Site (A5SS), Retained Intron (RI), Skipped Exon (SE).

Supplementary Data 2: Proteomic Analysis of Proteins Potentially Binding to SmD2.
