# Supplementary material for: Acetylation-dependent regulation of core spliceosome modulates hepatocellular carcinoma cassette exons and sensitivity to PARP inhibitors
Source: Nat Commun. 2024 Jun 18;15:5209. doi: 10.1038/s41467-024-49573-7 (PMC11189467; doi:10.1038/s41467-024-49573-7)
Supplement: Supplementary file 5 — Reporting Summary [file 41467_2024_49573_MOESM5_ESM.pdf]

nature portfolio

Corresponding author(s): Lianxin Liu

Last updated by author(s): Mar 28, 2024

Reporting Summary

Nature Portfolio wishes to improve the reproducibility of the work that we publish. This form provides structure for consistency and transparency in reporting. For further information on Nature Portfolio policies, see our Editorial Policies and the Editorial Policy Checklist.

Statistics

For all statistical analyses, confirm that the following items are present in the figure legend, table legend, main text, or Methods section.

n/a	Confirmed	
☐	☒	The exact sample size (*n*) for each experimental group/condition, given as a discrete number and unit of measurement
☐	☒	A statement on whether measurements were taken from distinct samples or whether the same sample was measured repeatedly
☐	☒	The statistical test(s) used AND whether they are one- or two-sided<br>*Only common tests should be described solely by name; describe more complex techniques in the Methods section.*
☐	☒	A description of all covariates tested
☐	☒	A description of any assumptions or corrections, such as tests of normality and adjustment for multiple comparisons
☐	☒	A full description of the statistical parameters including central tendency (e.g. means) or other basic estimates (e.g. regression coefficient) AND variation (e.g. standard deviation) or associated estimates of uncertainty (e.g. confidence intervals)
☐	☒	For null hypothesis testing, the test statistic (e.g. *F*, *t*, *r*) with confidence intervals, effect sizes, degrees of freedom and *P* value noted<br>*Give P values as exact values whenever suitable.*
☒	☐	For Bayesian analysis, information on the choice of priors and Markov chain Monte Carlo settings
☐	☒	For hierarchical and complex designs, identification of the appropriate level for tests and full reporting of outcomes
☐	☒	Estimates of effect sizes (e.g. Cohen's *d*, Pearson's *r*), indicating how they were calculated

Our web collection on statistics for biologists contains articles on many of the points above.

Software and code

Policy information about availability of computer code

Data collection	The RNA-seq libraries were sequenced using an Illumina sequencing platform, specifically the HiSeq™ 2500 or HiSeq X Ten systems, producing 125 bp or 150 bp paired-end reads. The raw data (raw reads) were subsequently processed with Trimmomatic.<br>MS/MS data were processed using MaxQuant search engine (v.1.6.15.0),searching tandem mass spectra against the human SwissProt database concatenated with the reverse decoy database.<br>Constitutive exons were obtained from HEXEvent, while cassette exons were identified using RNA-seq data. Reference alternative exons, which are not regulated by SmD2, were determined by excluding exons affected by SmD2 based on data from HEXEvent.
Data analysis	Publicly available software, such asGraphPad Prism (10.1.0) or R software (4.3.2), rMATS (v4.0.1), GSEA (v3.0), ViennaRNA (v2.5.0), ggseqlogo (PMID 29036507), and ggplot2 (v.3.3.3), were utilized in this study.

For manuscripts utilizing custom algorithms or software that are central to the research but not yet described in published literature, software must be made available to editors and reviewers. We strongly encourage code deposition in a community repository (e.g. GitHub). See the Nature Portfolio guidelines for submitting code & software for further information.

Data

Policy information about availability of data

All manuscripts must include a data availability statement. This statement should provide the following information, where applicable:

- Accession codes, unique identifiers, or web links for publicly available datasets
- A description of any restrictions on data availability
- For clinical datasets or third party data, please ensure that the statement adheres to our policy

RNA-seq data generated of this study have been deposited in the Genome Sequence Archive Human [HRA005004]. The Label-Free Quantitative Proteomics MS data are being deposited in PRIDE [PXD043544]. The MS data of immunoprecipitation against SmD2 are being deposited in PRIDE [PXD043535]. The dataset derived from this resource that supports the findings of this study is available at GEPIA2 (http://gepia2.cancer-pku.cn/). Regarding the TCGA datasets, these were obtained from UCSC XENA, available at https://xenabrowser.net/datapages/. Any additional data supporting this study's conclusions can be furnished by the corresponding authors upon a reasonable request.

Research involving human participants, their data, or biological material

Policy information about studies with human participants or human data. See also policy information about sex, gender (identity/presentation), and sexual orientation and race, ethnicity and racism.

Reporting on sex and gender	This study did not apply to just one gender, and we did not take gender into account in our study. We also did not apply the term gender in our article.
Reporting on race, ethnicity, or other socially relevant groupings	This study did not involve socially relevant categorical variables.
Population characteristics	HCC patient study participants were matched for age (range 37-79 years old), sex, and ethnicity in the discovery.
Recruitment	Label-free quantitative proteome analysis and Sm proteins expression analysis were performed on specimens from HCC patients undergoing surgery at the First Affiliated Hospital of USTC between June and December 2021. PDX models were established using the specimen procured from one HCC patient scheduled for surgical treatment in September 2022. Informed consent was acquired from all participants, and the study received approval from the Institutional Review Board. Two HCC tissue microarrays (TMAs) were used, with TMA1 containing 48 formalin-fixed, paraffin-embedded HCC tissues and TMA2 consisting of 58 HCC tumor and adjacent normal tissues. These samples were collected from patients who underwent curative resection at the Department of Hepatobiliary Surgery, First Affiliated Hospital of USTC.
Ethics oversight	Informed consent was obtained from the patients who provide HCC specimens, and the research was approved by the Ethics Committee of the First Affiliated Hospital of USTC, the approval number is 2022-KY293. This statement has been included in the Methods section of our manuscript.

Note that full information on the approval of the study protocol must also be provided in the manuscript.

Field-specific reporting

Please select the one below that is the best fit for your research. If you are not sure, read the appropriate sections before making your selection.

[x] Life sciences   [ ] Behavioural & social sciences   [ ] Ecological, evolutionary & environmental sciences

For a reference copy of the document with all sections, see nature.com/documents/nr-reporting-summary-flat.pdf

Life sciences study design

All studies must disclose on these points even when the disclosure is negative.

Sample size

Our HCC sample selection originated from the hepatocellular carcinoma sample library at the First Affiliated Hospital of the University of Science and Technology of China. These samples were randomly selected without specific inclusion criteria to minimize selection bias and better represent a broad patient demographic. This approach ensures that our findings can be generalized to a wider HCC patient population.

Initially, Sm protein was identified as the primary research target from a preliminary screening of samples from 6 patients. Subsequently, we analyzed the expression of SmD2 in 105 pairs of HCC samples. The decision on this sample size was influenced by the availability of samples from the library and the intention to achieve a statistically meaningful analysis of SmD2 expression variability. Although no pre-defined sample size calculation was performed, the chosen sample size is deemed sufficient based on prior research experiences and the capacity of our sample library, allowing for significant statistical analysis and conclusions.

For in vitro experiments, we did not conduct a predetermined sample size calculation but opted for independent experiments with n=3, incorporating at least 3 biological replicates and, where possible, technical replicates for each experiment. This protocol is based on laboratory norms and preliminary experiment outcomes, considered adequate for ensuring the reproducibility and reliability of our results.

In our animal studies, we ensured a minimum of 5 mice per group. This decision was guided by preliminary data and standard practices in the

literature, aiming to balance statistical power with considerations of animal welfare.

In summary, our sample size choices were informed by prior research experience, the availability of samples, and the requirements of our experimental design. Despite the absence of rigorous pre-study sample size calculations, we believe our approach, combined with careful experimental design and data analysis, supports the validity of our research objectives and conclusions.

Data exclusions	No data were excluded from analysis.
Replication	Each experiment was conducted a minimum of three times to ensure reproducibility. Additionally, to account for biological variability, we used multiple independent samples for our assays. Where applicable, experiments were also independently replicated by different team members to confirm the consistency of the results.
Randomization	All samples were randomly assigned to various groups.
Blinding	In our study, blinding was not applicable as it is exploratory in nature and does not involve any elements that could be influenced by bias from the subject or observer.

Reporting for specific materials, systems and methods

We require information from authors about some types of materials, experimental systems and methods used in many studies. Here, indicate whether each material, system or method listed is relevant to your study. If you are not sure if a list item applies to your research, read the appropriate section before selecting a response.

Materials & experimental systems

n/a	Involved in the study	
☐	☒	Antibodies
☐	☒	Eukaryotic cell lines
☒	☐	Palaeontology and archaeology
☐	☒	Animals and other organisms
☒	☐	Clinical data
☒	☐	Dual use research of concern
☒	☐	Plants

Methods

n/a	Involved in the study	
☒	☐	ChIP-seq
☒	☐	Flow cytometry
☒	☐	MRI-based neuroimaging

Antibodies

Antibodies used

The antibodies were as follows: anti-Ack rabbit antibody (9441s, 1:800 for Immunoblot), anti-HA mouse mAb (2367, 1:1000 for Immunoblot and 1:100 for immunofluorescence), anti-HA rabbit mAb (3724, 1:1000 for Immunoblot), anti-HDAC2 rabbit mAb (57156, 1:1000 for Immunoblot), anti-Poly/Mono-ADP Ribose rabbit mAb (83732, 1:1000 for Immunoblot), anti-p300 rabbit mAb (86377, 1:1000 for Immunoblot and 1:300 for immunofluorescence), anti-Flag rabbit mAb (14793, 1:1000 for Immunoblot), anti-CBP rabbit mAb (7389, 1:1000 for Immunoblot), anti-Vinculin rabbit mAb (13901, 1:1000 for Immunoblot), anti-BRCA1 rabbit mAb (9010, 1:1000 for Immunoblot and 1:100 for IHC), anti-β-Actin mouse mAb (3700, 1:1000 for Immunoblot), anti-PARP1 rabbit mAb (9532, 1:1000 for Immunoblot and 1:50 for IHC) and Rabbit mAb IgG Isotype Control (3900) are from Cell Signaling Technology. Anti-SmD2 antibody rabbit antibody (198296, 1:1000 for Immunoblot and I:200 for IHC) and anti-gamma H2A.X (phospho S139) rabbit antibody (81299, 1:1000 for Immunoblot, 1:500 for immunofluorescence, and I:200 for IHC) are from Abcam. Anti-FANCA rabbit antibody (11975-1-AP, 1:800 for Immunoblot and 1:50 for IHC) and anti-FANCD2 rabbit antibody (28619-1-AP, 1:800 for Immunoblot and 1:50 for IHC) are from Proteintech. Alexa Fluor 488 conjugated goat anti-mouse IgG (A11001), Alexa Fluor 488 conjugated goat anti-rabbit IgG (A11008). Alexa Fluor 555 conjugated goat anti-mouse IgG (A21422), Alexa Fluor 555 conjugated goat anti-rabbit IgG (A21428) and Alexa Fluor™ 555 Phalloidin (A34055) are from Thermo Fisher Scientific.

Validation

anti-Ack rabbit antibody (9441s, https://www.cellsignal.com/products/primary-antibodies/acetylated-lysine-antibody/9441),
anti-HA mouse mAb (2367, https://www.cellsignal.com/products/primary-antibodies/ha-tag-6e2-mouse-mab/2367),
anti-HA rabbit mAb (3724, https://www.cellsignal.com/products/primary-antibodies/ha-tag-c29f4-rabbit-mab/3724),
anti-HDAC2 rabbit mAb (57156, https://www.cellsignal.com/products/primary-antibodies/hdac2-d6s5p-rabbit-mab/57156),
anti-Poly/Mono-ADP Ribose rabbit mAb (83732, https://www.cellsignal.com/product/productDetail.jsp?productId=83732),
anti-p300 rabbit mAb (86377, https://www.cellsignal.com/products/primary-antibodies/p300-d8z4e-rabbit-mab/86377), anti-Flag rabbit mAb (14793, https://www.cellsignal.com/products/primary-antibodies/dykddddk-tag-d6w5b-rabbit-mab-binds-to-same-epitope-as-sigma-aldrich-anti-flag-m2-antibody/14793),
anti-CBP rabbit mAb (7389, https://www.cellsignal.com/products/primary-antibodies/cbp-d6c5-rabbit-mab/7389),
anti-Vinculin rabbit mAb (13901, https://www.cellsignal.com/products/primary-antibodies/vinculin-e1e9v-xp-174-rabbit-mab/13901),
anti-BRCA1 rabbit mAb (9010, https://www.cellsignal.com/products/primary-antibodies/brca1-antibody/9010),
anti-β-Actin mouse mAb (3700, https://www.cellsignal.com/products/primary-antibodies/b-actin-8h10d10-mouse-mab/3700),
anti-PARP1 rabbit mAb (9532, https://www.cellsignal.com/products/primary-antibodies/parp-46d11-rabbit-mab/9532)
Rabbit mAb IgG Isotype Control (3900, https://www.cellsignal.com/products/primary-antibodies/rabbit-da1e-mab-igg-xp-174-isotype-control/3900)

Anti-SmD2 antibody rabbit antibody (198296, https://www.abcam.com/products/primary-antibodies/snrpd2-antibody-epr16762-

ab198296.html)
anti-gamma H2A.X (phosphoS139) rabbit antibody (81299, https://www.abcam.com/products/primary-antibodies/gamma-h2ax-phospho-s139-antibody-ep8542y-ab81299.html)
Anti-FANCA rabbit antibody (11975-1-AP, https://www.ptgcn.com/products/FANCA-Antibody-11975-1-AP.htm)
anti-FANCD2 rabbit antibody (28619-1-AP, https://www.ptgcn.com/products/FANCD2-Antibody-28619-1-AP.htm)

Alexa Fluor 488 conjugated goat anti-mouse IgG (A11001, https://www.thermofisher.cn/cn/zh/antibody/product/Goat-anti-Mouse-IgG-H-L-Cross-Adsorbed-Secondary-Antibody-Polyclonal/A-11001),
Alexa Fluor 488 conjugated goat anti-rabbit IgG (A11008, https://www.thermofisher.cn/cn/en/antibody/product/Goat-anti-Rabbit-IgG-H-L-Cross-Adsorbed-Secondary-Antibody-Polyclonal/A-11008).
Alexa Fluor 555 conjugated goat anti-mouse IgG (A21422, https://www.thermofisher.cn/cn/en/antibody/product/Goat-anti-Mouse-IgG-H-L-Cross-Adsorbed-Secondary-Antibody-Polyclonal/A-21422),
Alexa Fluor 555 conjugated goat anti-rabbit IgG (A21428, https://www.thermofisher.cn/cn/en/antibody/product/Goat-anti-Rabbit-IgG-H-L-Cross-Adsorbed-Secondary-Antibody-Polyclonal/A-21428)
Alexa Fluor™ 555 Phalloidin (A34055, https://www.thermofisher.cn/order/catalog/product/A34055)

Eukaryotic cell lines

Policy information about cell lines and Sex and Gender in Research

Cell line source(s)	HCCLM3, Huh7, PLC/PRF/5, Hepa1-6, LX-2 and HEK293T cells were cultured in the DMEM (Sigma), which contained 10% FBS (Sigma), 100 U penicillin (Gibco) and 100 ug ml-1 streptomycin (Gibco). PLC/PRF/5 and Huh7 were purchased from Accegen. Hepa1-6 and LX-2 were gifts from Prof. Qingsong Hu (USTC). HEK293T was purchased from ATCC. HCCLM3 was a gift from Prof. Hongyang Wang (SMMU).
Authentication	The cells were submitted for short tandem repeat (STR) profiling to confirm their authenticity.
Mycoplasma contamination	These cell lines were tested negative.
Commonly misidentified lines (See ICLAC register)	No commonly misidentified cell lines were used.

Animals and other research organisms

Policy information about studies involving animals; ARRIVE guidelines recommended for reporting animal research, and Sex and Gender in Research

Laboratory animals	In this series of experiments, we utilized adult male and female Alb-Cre mice, H11-LSL-Myc mice, NSG mice (sourced from Shanghai Model Organisms Center), wild-type C57BL/6 mice, and male nude mice (obtained from Shanghai SLAC Laboratory Animal) aged between 5 to 12 weeks and weighing 20 to 28 grams. These mice were maintained in an environment with controlled temperature (22±1°C), approximately 50% humidity, and a 12-hour light/12-hour dark cycle, with lights on from 6 am (ZT0) to 6 pm. Group housing was implemented for the mice, ranging from 2 to 5 per group, and they were given unrestricted access to food and water.
Wild animals	This study does not include wild animals.
Reporting on sex	The findings of this study are not limited to a specific sex or gender. Additionally, no data regarding sex or gender were collected in this research. Only a portion of the experiments, such as the application of xenografts and spontaneous tumor formation in mice, involved male mice. Both NSG mice and C57BL/6 mice with MYC-driven HCC included both male and female mice.
Field-collected samples	Study didn't involve samples collected in the field.
Ethics oversight	Animal ethical guidelines were observed and approved by the Animal Ethics Committee of the First Affiliated Hospital of USTC for all animal experiments, the approval number is 2022-N(A)-084.

Note that full information on the approval of the study protocol must also be provided in the manuscript.
